# Plasmolipin deficiency is essential for HUVECs survival under hypoxic conditions

**DOI:** 10.1038/s41420-025-02526-5

**Published:** 2025-05-17

**Authors:** Yanghua Li, Weiling Man, Xiang Li, Xiaojie Wu, Yumeng Cui, Shiyun Chen, Xianhong Li, Yanli Lin, Lihe Jiang, Youliang Wang

**Affiliations:** 1 Medical College, Guangxi University, Nanning, China; 2 Laboratory of Advanced Biotechnology, Beijing Institute of Biotechnology, Beijing, China; 3 School of Basic Medical Sciences, Youjiang Medical University for Nationalities, Baise, China; 4 Guangdong Provincial Key Laboratory of Chemical Genomics, Peking University Shenzhen Graduate School, Shenzhen, China

**Keywords:** Cell biology, Molecular biology

## Abstract

This study aims to explore the molecules that affect the survival of Human Umbilical Vein Endothelial Cells (HUVECs) under hypoxia and their mechanisms of action. In hypoxia, plasmolipin (PLLP) was identified through the screening of CRISPR/Cas9 and small guide RNA (sgRNA) library. Functionally, PLLP knockout led to increase cell proliferation, cellular metabolism, tight junction formation, angiogenesis ability, migration and invasion in hypoxic HUVECs. Furthermore, PLLP knockout countered the inhibitory effects of bevacizumab on HUVECs angiogenesis and cell survival in hypoxic conditions. PLLP knockout was found to modulate the survival of HUVECs in hypoxia by enhancing the phosphorylation of AKT and ERK1/2 proteins. In conclusion, inhibiting the expression of PLLP in HUVECs promotes cell survival and maintenance of cellular functions under hypoxic condition. PLLP plays a crucial role in regulating cell survival in hypoxia through the activation of AKT and ERK1/2 pathways. This study identifies novel molecules that affect HUVECs survival under hypoxic conditions and provides a new possibility for future studies on cell survival under hypoxic conditions.

## Introduction

Endothelial cells (ECs) lining the capillaries of the vascular system are essential for delivering oxygen and nutrients to various parts of the body [1]. In healthy adults, these quiescent ECs, equipped with oxygen sensors and hypoxia-inducible factors, have a long life [2, 3]. In response to pathological conditions, ECs can trigger the formation of new vessels to facilitate the delivery of oxygen and nutrients to hypoxic tissues, leading to a change in the metabolic pathway of ECs [4]. Human umbilical vein endothelial cells (HUVECs) serve as a representative in vitro model for studying EC function and are widely employed to investigate the molecular mechanisms of angiogenesis [5]. Furthermore, HUVECs are utilized in research areas such as hypoxia, inflammation, oxidative stress, and tissue engineering [6–8]. Vascular endothelial growth factor (VEGF) is a critical activator of angiogenesis that not only inhibits the apoptotic capacity of HUVECs, but also enhances vascular permeability and promotes cell migration [9, 10].

Hypoxia, defined as the absence of endogenous oxygen bound to cells at levels below physiological oxygen tension, plays a critical role in both physiological and pathological processes. Inadequate oxygen supply or disrupted oxygen consumption result in detrimental shifts in tissue metabolism, function, and morphological structure [11–13]. The cellular response to hypoxia involves intricate signaling pathways, including the HIF, PI3K/AKT, MAPK, and NF-κB pathways, which create positive and negative feedback loops enhancing or mitigating the effects of hypoxia [14–16]. Within an anoxic microenvironment, there are alterations in cellular survival states and metabolic pathways leading to changes in the reactions of endothelial cells, such as reduced cell proliferation [17]. Given the complexity of cellular reactions to hypoxia, therapeutic interventions in clinical settings currently have limited effectiveness. To better understand these complex responses, we employed HUVECs as a cellular model to investigate the impact of hypoxic conditions on endothelial cell survival. HUVECs exhibit a relatively well-defined response mechanism to hypoxia; for example, they significantly upregulate HIF-1α and its downstream target genes, playing a crucial role in angiogenesis and metabolic adaptation [18]. Furthermore, HUVECs are derived from the umbilical cord, and their hypoxic response closely mimics that of endothelial cells in human diseases. The stable and reproducible response of HUVECs to hypoxia under laboratory conditions makes them particularly suitable for mechanistic studies and drug screening [19, 20]. In this study, we utilized immortalized HUVECs, a widely used cell model for studying endothelial cells [21, 22], to screen for hypoxia-related genes. The identification of these genes may provide insights into the mechanisms underlying cellular responses to hypoxia and potentially expand the range of effective therapeutic interventions.

The Clustered Regularly Interspaced Short Palindromic Repeats (CRISPR)/Cas9 system, an adaptive immune system evolved from bacteria and archaea to defend against foreign viruses and DNA, plays a crucial role in recognition when an organism is invaded by foreign DNA fragments [23, 24]. This system has been adapted for genome-wide screening applications, offering an alternative to RNA interference for elucidating novel biological mechanisms across diverse models [23, 25, 26]. The CRISPR/Cas9 gene editing technique, guided by single-stranded guide RNAs (sgRNAs), enables gene disruption in eukaryotic cells [27]. Specifically, the introduction of a genome-scale CRISPR-Cas9 knockout (GeCKO) library into human cells facilitates the simultaneous knockout of multiple genes across different cells. This allows for the screening of genes that influence cell survival under defined conditions. The GeCKO v2 library, comprising 122,417 unique sgRNAs targeting 19,052 genes, permits both negative and positive selection screens upon transfection into human cells [28]. GeCKO achieves functional loss-of-function mutations in genomic DNA; the resulting homozygous knockouts can yield high screening sensitivity, which is particularly valuable when a knockout does not fully abolish gene function [27]. In this study, we employed a genome-wide CRISPR knockout (GeCKO) library to identify mutations affecting cell function in hypoxic environments. By utilizing the GeCKO v2 library to screen for hypoxia resistance genes in HUVECs, this research aims to provide insights into cellular survival and the corresponding molecular mechanisms under hypoxic conditions. Through this CRISPR/Cas9 and GeCKO v2 library-based hypoxia screening, we identified the hypoxia-related gene plasmolipin (PLLP).

PLLP, a proteolipid encoded by the MAL gene, is predominantly expressed in the nervous system, particularly in oligodendrocytes, myelin sheaths, and myelinated tracts of the central nervous system (CNS). As a proteolipid protein belonging to the tetraspan molecule family, PLLP is also found in epithelial cells of various organs, including the kidneys, intestines, and brain [29, 30]. Within the nervous system, its localization includes the brain, cerebellum, oligodendrocyte membranes, and myelin sheaths [31–33]. Given this established expression pattern, this study aims to preliminarily investigate the effect of PLLP on HUVEC under hypoxic conditions and to explore key factors influencing endothelial cell survival. This is the first report, to our knowledge, examining the effects of PLLP on HUVEC proliferation, apoptosis, migration, metabolism, and angiogenesis under hypoxia. The results of this study offer a preliminary exploration of that PLLP influences HUVEC survival in hypoxia by AKT or ERK signaling pathways, and PLLP influences the inhibitory effect of bevacizumab under hypoxic conditions, potentially via modulation of the AKT or ERK signaling pathways. The discovery potentially providing novel insights for future research on cellular responses to oxygen deprivation.

## Results

### Genome-wide CRISPR/Cas9 screened the key hypoxia tolerant gene PLLP

**Fig. 1:**
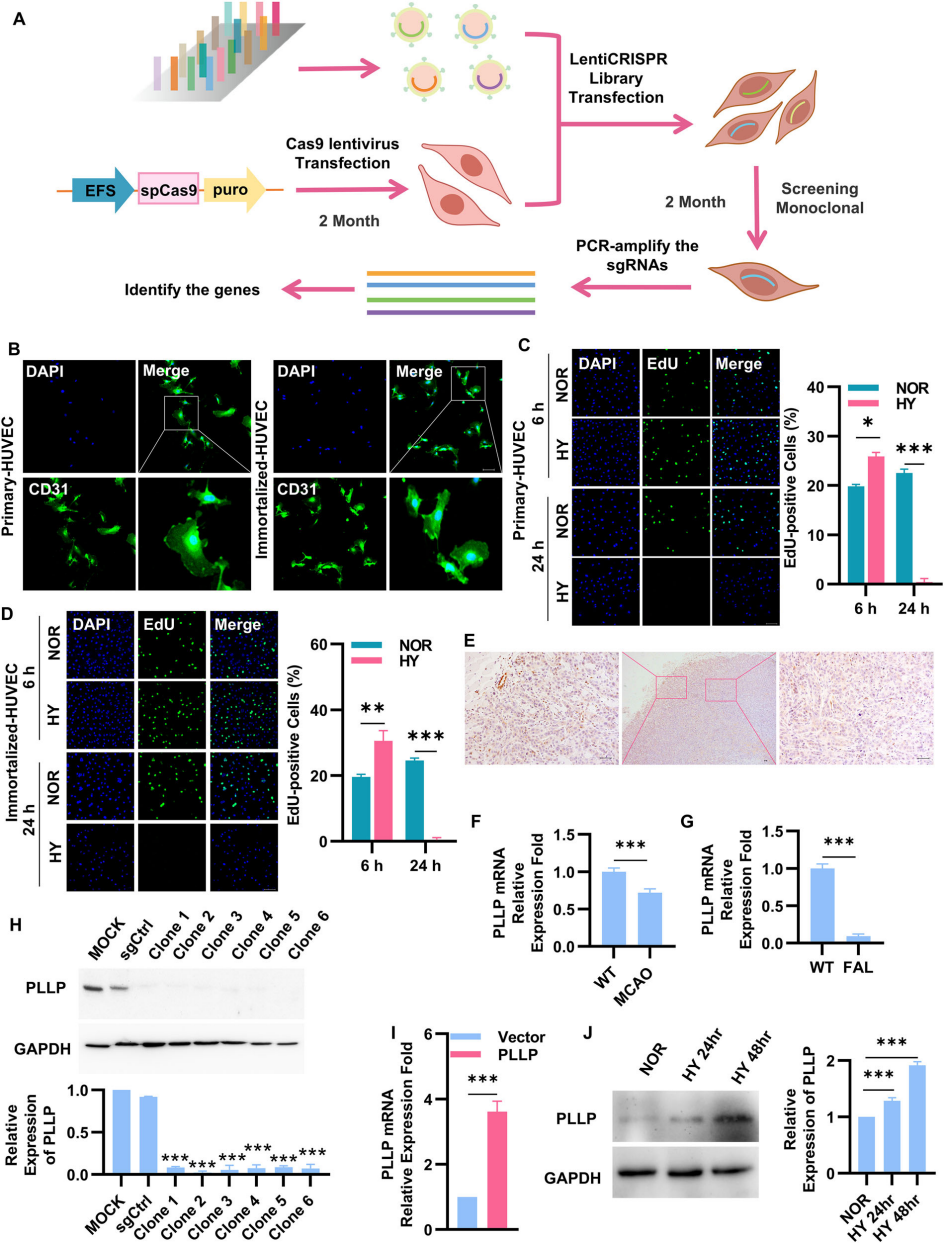
Screening of hypoxia related factors in HUVEC cells. **A** Schematic diagram of screening for hypoxia related factors in HUVECs. **B** Primary HUVECs and immortalized HUVECs immunostaining showed CD31 (green). **C** EdU incorporation experiment to detect the cell proliferation ability of primary HUVECs under normal and hypoxic conditions for 6 hours and 24 hours. NOR, cells cultured in normoxia. HY, cells cultured in hypoxia. Blue represents DAPI, represents total cells, green represents EdU, represents proliferative cells. Scale bar, 25 μm. **D** EdU incorporation experiment to detect the cell proliferation ability of immortalized HUVECs under normal and hypoxic conditions for 6 h and 24 h. Blue represents DAPI, represents total cells, green represents EdU, represents proliferative cells. Scale bar, 25 μm. **E** IHC staining of PLLP in xenograft tumors. The scale bars are 25 μm. **F** mRNA expression of PLLP in cerebral ischemic models (*n* = 6). **G** mRNA expression of PLLP in hindlimb ischemic models (*n* = 6). **H** Protein expression of PLLP after HUVECs transfected PLLP-gRNA. MOCK, no transfected HUVECs. sgCtrl, the negative control group of PLLP knockout cells. **I** mRNA expression of PLLP after HUVECs overexpressed PLLP. Vector, the negative control group of PLLP overexpressing cells. PLLP, the PLLP overexpressing cells. **J** Protein expression of PLLP in HUVECs after 24 or 48 hours in hypoxia. The results are expressed as the mean ± standard deviation and analyzed using a two-tailed Student’s t-test (*n* = 3, *p < 0.05, **p < 0.01, ***p < 0.001, ns=not significant).

HUVECs were chosen as the experimental model for screening genes involved in hypoxia-induced cell death (Fig. 1A). To facilitate multiple transfections and monoclonal cell selection, we chose immortalized HUVECs [34, 35]. We confirmed that these immortalized HUVECs expressed CD31, similar to primary HUVECs, thereby demonstrating that they share the same cellular characteristics (Fig. 1B). Furthermore, the expression levels of other endothelial-related factors were found to be comparable between primary and immortalized HUVECs (Fig. S1A). Under short-term (6 h) hypoxia, the proliferation of primary HUVEC increased slightly, while long-term (24 h) severe hypoxia exposure resulted in significant morphological changes and decreased proliferation cell (Fig. 1C). Immortalized cells exhibited similar proliferation characteristics under these conditions (Fig. 1D), highlighting their sensitivity to severe hypoxia. The above results confirmed that primary and immortalized HUVEC exhibit similar proliferation characteristics under hypoxia, justifying the use of immortalized HUVECs for further research. Initially, lentiviral Cas9 was transfected into HUVECs to generate eight monoclonal cells that stably expressed Cas9 at the genetic, and transcriptional and translational levels (Fig. S1B–D). Subsequent research aimed to detect the effect of hypoxia on the growth of control cells by exposing HUVECs and control-gRNA cells to both normal and hypoxic conditions. Hypoxia exposure led to obvious morphological changes and increased cell death, which confirmed that HUVECs transfected with Cas9 could serve as an effective negative control (Fig. S1E). Following this, the GeCKO v2 library was transfected into Cas9 cells, leading to the identification of three monoclonal cells that were able to survive and grow normally under hypoxic conditions. By sequencing the PCR products from the genome of these surviving cells, sgRNAs integrated into the genome were identified. Comparative analyses with existing databases revealed the presence of PLLP-gRNA (PLLP-gRNA) existed in the genome of in one of the monoclonal cells, indicating that the gRNA of PLLP resided in the first exon of PLLP (Fig. S1F). To investigate the role of PLLP in hypoxic conditions, we examined its expression in vivo using several models. First, analysis of lung xenograft tumors from our previous study [36] revealed differential PLLP expression in endothelial cells. Specifically, PLLP was highly expressed in endothelial cells at the periphery of the tumor, where oxygen concentration is high, but exhibited significantly lower expression in endothelial cells within the hypoxic core of the tumor (Fig. 1E). This observation suggests that PLLP downregulation under hypoxia may promote endothelial cell survival. Consistent with this, we also observed a significant downregulation of PLLP expression in both cerebral ischemia and hindlimb ischemia mouse models (Fig. 1F, G). Subsequently, PLLP-gRNA was introduced into HUVECs via lentivirus, and monoclonal cells were screened again. In this process, primers targeting sequences from exon 2 to exon 3 showed a significant reduction in PLLP expression at the mRNA level in monoclonal cells post-insertion of PLLP-gRNA (Fig. S1I). Western blot analysis illustrated a substantial decrease in PLLP protein expression (Fig. 1H). Introduction of gRNA induced the insertion or deletion (Indel) of bases resulting in nonsense mutations. The nonsense-mediated decay (NMD) of mRNA could activate mRNA quality control (QC) event to remove the defective mRNAs leading to premature termination of protein translation [37–39]. PCR products sequencing revealed six distinct mutations in the base sequence (Fig. S1G). Since HUVECs are derived from umbilical cord, we also additionally verified their cell proliferation in physiological oxygen environment and in vitro culture oxygen environment. It can be seen from the results that there is no significant difference in the cell proliferation of HUVECs in 21% O_2_ and 6% O_2_ environments (Fig. S1H). Therefore, in subsequent cell culture, we continued to use the conventional oxygen concentration of cells cultured in vitro, that is, 21% O_2_ as normoxic conditions [40–42]. Moreover, HUVECs were transfected with lentiviruses overexpressing PLLP, resulting in stable overexpression of PLLP, with subsequent verification of protein and mRNA levels (Fig. 1I and Fig. S1J). Under hypoxic conditions, PLLP expression in HUVECs was significantly upregulated, suggesting a link between PLLP and HUVEC survival during hypoxia (Fig. 1J and Fig. S1K), thereby indicating that increased PLLP expression correlated with decreased cell survival under hypoxic conditions. Additionally, analysis of mouse tissues demonstrated elevated PLLP expression in endothelium-rich tissues (Fig. S2A and Fig. S2B).

### Knockout of PLLP is essential to HUVECs survival in hypoxia

**Fig. 2:**
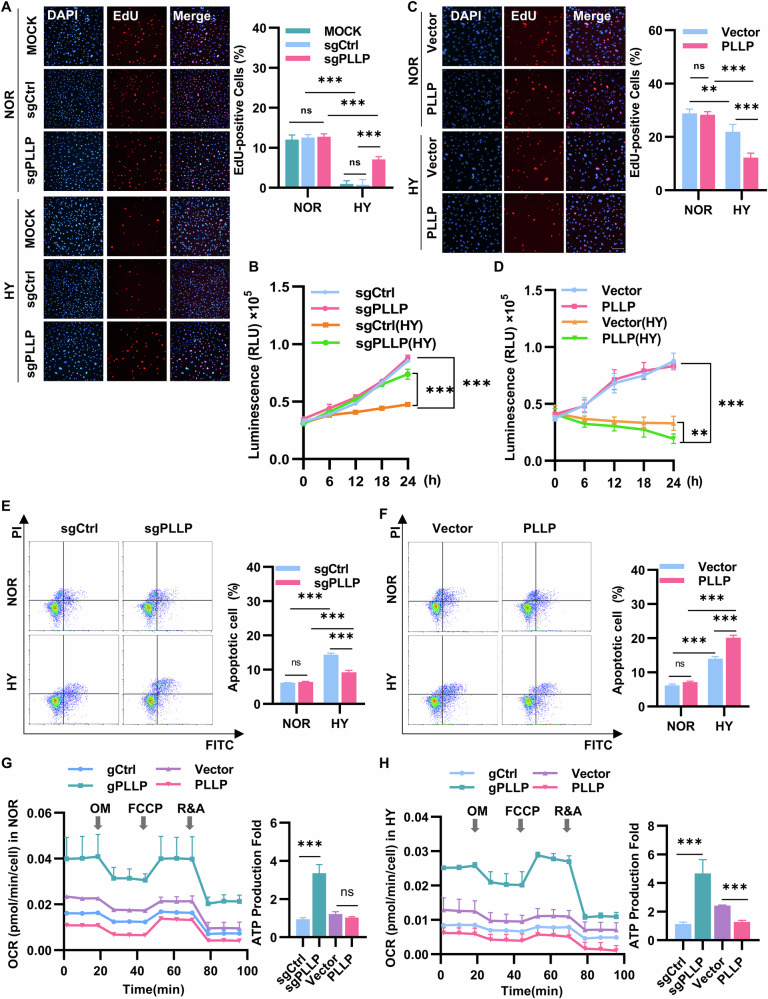
Knockout of PLLP increased the survival ability of HUVECs under hypoxic conditions. **A** EdU incorporation experiment to detect the cell proliferation ability of sgPLLP and sgCtrl cells under normal and hypoxic conditions for 24 hours. MOCK, no transfected HUVECs. sgCtrl, the negative control group of PLLP knockout cells. sgPLLP, the PLLP knockout cells. Blue represents DAPI, represents total cells, red represents EdU, represents proliferative cells. Scale bar, 25 μm. **B** Used CellTiter-Glo to analyze the cell viability of sgCtrl and sgPLLP cells in normal and hypoxia at 0, 6, 12, 18, and 24 hours. **C** EdU incorporation experiment to detect cell proliferation ability of PLLP and Vector cells cultured under normal and hypoxic conditions for 12 hours. Vector, the negative control group of PLLP overexpressing cells. PLLP, the PLLP overexpressing cells. Scale bar, 25 μm. **D** Used CellTiter-Glo to analyze the cell viability of Vector and PLLP cells in normal and hypoxia at 0, 6, 12, 18, and 24 hours. **E** Flow cytometry analysis of sgCtrl or sgPLLP cells cultured in normal and hypoxia for 48 hours. **F** Flow cytometry analysis of Vector and PLLP cultured in normal and hypoxia for 24 hours. The results are expressed as the mean ± standard deviation. **G** Mitochondrial stress test to detect OCR of HUVECs cultured under normal conditions for 24 hours. **H** Mitochondrial stress test to detect OCR of HUVECs cultured under hypoxic conditions for 24 hours. The results are expressed as the mean ± standard deviation. **A**–**F** analyzed using Two-way ANOVA and **G**, **H** analyzed using a two-tailed Student’s t-test (*n* = 3, *p < 0.05, **p < 0.01, ***p < 0.001, ns=not significant.).

To delve deeper into the role of PLLP in hypoxia tolerance, we investigated the impact of PLLP knockout on HUVECs viability. To determine whether PLLP knockdown affects the proliferation of primary HUVECs, we proceeded with the knockdown using siRNA, given the limitations associated with primary HUVECs (Fig. S3A). We then assessed the proliferation of these cells under hypoxic conditions and observed a significant decrease in their proliferation. Interestingly, upon knocking down PLLP, there was a notable increase in the proliferation of primary HUVECs (Fig. S3B). By detecting the proliferation of immortalized HUVEC, revealing that under normoxic conditions, deleting PLLP did not affect cell proliferation, as demonstrated in Fig. 2A. In contrast, when exposed to hypoxic conditions, the proliferation of control cells was hindered, whereas the proliferation of PLLP knockout cells remained stable (Fig. 2A), suggesting that PLLP deficiency can counteract the suppressive influence of hypoxia on HUVECs proliferation. Primary and immortalized HUVEC exhibit identical proliferation characteristics under both normal oxygen and hypoxia conditions after PLLP knockdown, which confirms the feasibility of using immortalized HUVEC for further research. Moreover, there was no significant difference in cell proliferation between HUVECs without gene modification and sgCtrl transfected with Cas9 in normoxia or hypoxia, so sgCtrl used as a negative control in subsequent experiments. Conversely, overexpressing PLLP did not alter cell proliferation under normoxic conditions, however, cell proliferation was impeded in PLLP-overexpressing cells under hypoxia (Fig. 2C). Notably, under hypoxic conditions, the viability of gPLLP cells surpassed that of sgCtrl cells (Fig. 2B). Similarly, the viability of HUVECs overexpressing PLLP decreased under hypoxia (Fig. 2D). These results emphasize that PLLP knockout mitigates hypoxia-induced damage on HUVECs viability, while PLLP overexpression exacerbates such detrimental effects. Flow cytometry analysis revealed no significant difference in the apoptosis rate between sgPLLP and sgCtrl under normoxic conditions. However, under hypoxic conditions, the apoptosis rate in sgPLLP cells was significantly lower than that in sgCtrl cells (Fig. 2E). In line with expectations, overexpression of PLLP did not impact apoptosis under normoxic conditions; nevertheless, apoptosis increased in PLLP-overexpressing cells under hypoxia (Fig. 2F).

Our study revealed that the viability of PLLP knockout cells increased under hypoxic conditions, prompting further investigation into the alterations in energy metabolism of cells. Through the oxygen consumption rate (OCR) test, we observed that mitochondrial respiration in PLLP knockout cells was significantly enhanced when exposed to hypoxia (Fig. 2G and Fig. S3C). Conversely, under identical conditions, cells overexpressing the PLLP exhibited weakened mitochondrial respiration (Fig. 2H and Fig. S3D). These outcomes indicate that PLLP knockout confers resistance to hypoxia-induced damage on HUVECs survival, highlighting the essential role of PLLP absence for cell survival in hypoxic environments, and also suggested a critical role of the PLLP in regulating cellular energy metabolism under hypoxia environments.

### PLLP deficiency is crucial for HUVECs migration and invasion in hypoxia

**Fig. 3:**
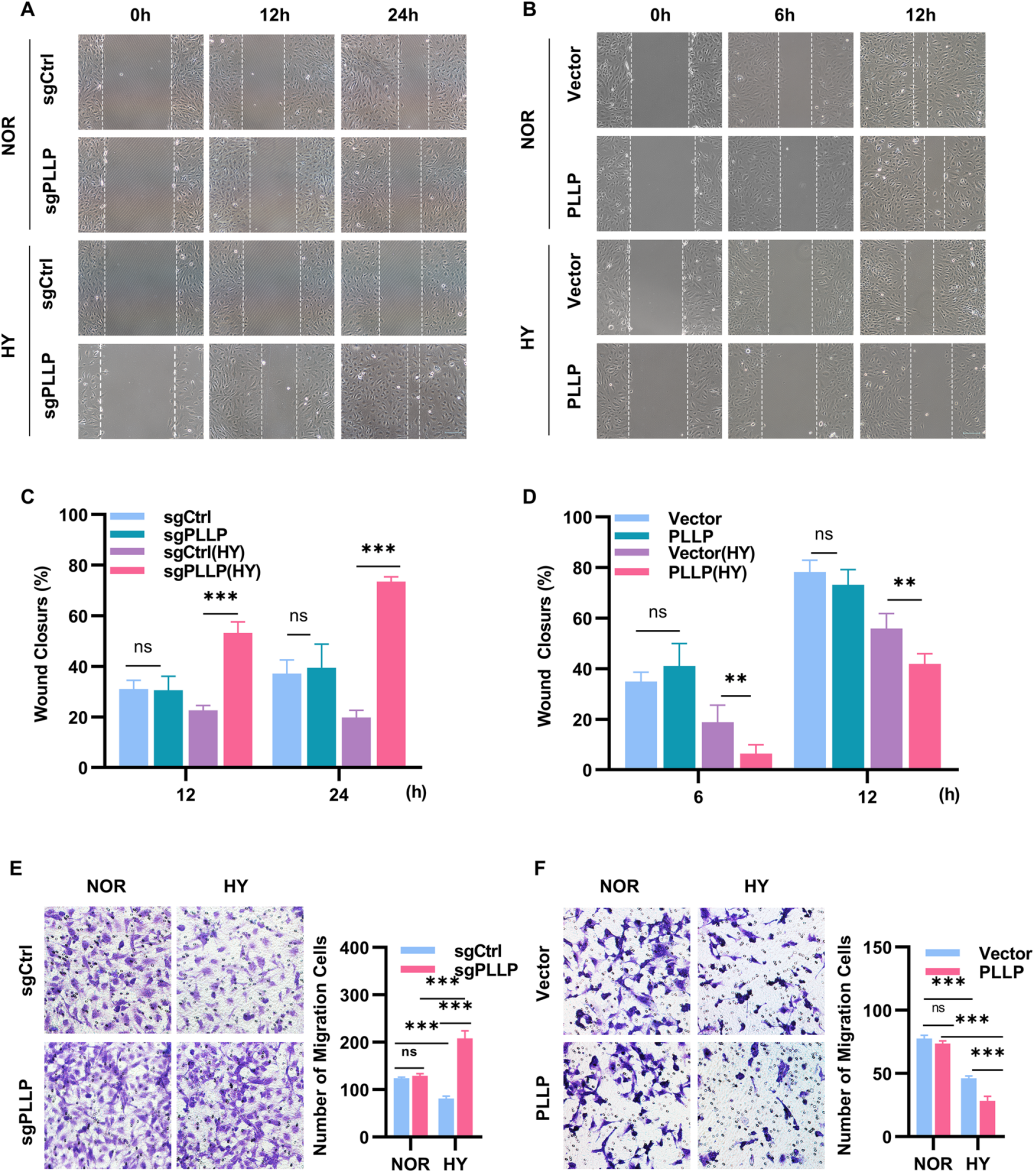
PLLP knockout increased the migration ability of HUVECs in hypoxia. **A**, **B** Wound healing assay to detect the migration ability of HUVECs after knocking out PLLP under normal and hypoxic conditions. **C**, **D** Wound healing assay to detect the migration ability of HUVECs after overexpressing PLLP under normal and hypoxic conditions. **E** Transwell migration assay to detect the migration ability of HUVEC cells with PLLP knocked out after 24 hours of normal and hypoxic culture. **F** Transwell migration assay to detect the migration ability of HUVEC cells with PLLP overexpressing after 24 hours of normal and hypoxic culture. The results are expressed as the mean ± standard deviation. **C**–**F** analyzed using Two-way ANOVA (*n* = 3, *p < 0.05, **p < 0.01, ***p < 0.001, ns not significant).

To further investigate the impact of PLLP on the migration and invasion abilities of HUVECs under hypoxic conditions, we conducted experiments to assess the effects of PLLP on EC cell migration and invasion under both normoxic and hypoxic conditions using wound healing and transwell assays. The results of the wound healing assay revealed that HUVECs lacking PLLP did not exhibit any significant differences in migration speed compared to the negative control cells under normoxic conditions (Fig. 3A, B). However, the migration speed of HUVECs with sgPLLP was notably higher than that of cells with sgCtrl under hypoxic conditions (Fig. 3A, B). Conversely, the migration speed of PLLP-overexpressing cells under hypoxia was significantly lower than that of the vector control (Fig. 3C,D). Furthermore, the transwell migration experiment results showed that the migration rate of cells with sgPLLP was significantly higher than that of cells with sgCtrl under hypoxic conditions (Fig. 3E), whereas the migration rate of cells overexpressing PLLP was significantly lower than that of the vector control (Fig. 3F). Additionally, the invasion rate of HUVECs with sgPLLP was significantly higher than that of cells with sgCtrl (Fig. S4A) under hypoxia, while the PLLP overexpressing group showed a lower invasion rate compared to the control group under hypoxic conditions (Fig. S4B). Overall, these findings suggest that under hypoxic conditions, the knockout of PLLP enhances the migration and invasion abilities of HUVECs.

### The absence of PLLP enabled HUVECs to have the ability to angiogenesis and tight junctions in hypoxia

**Fig. 4:**
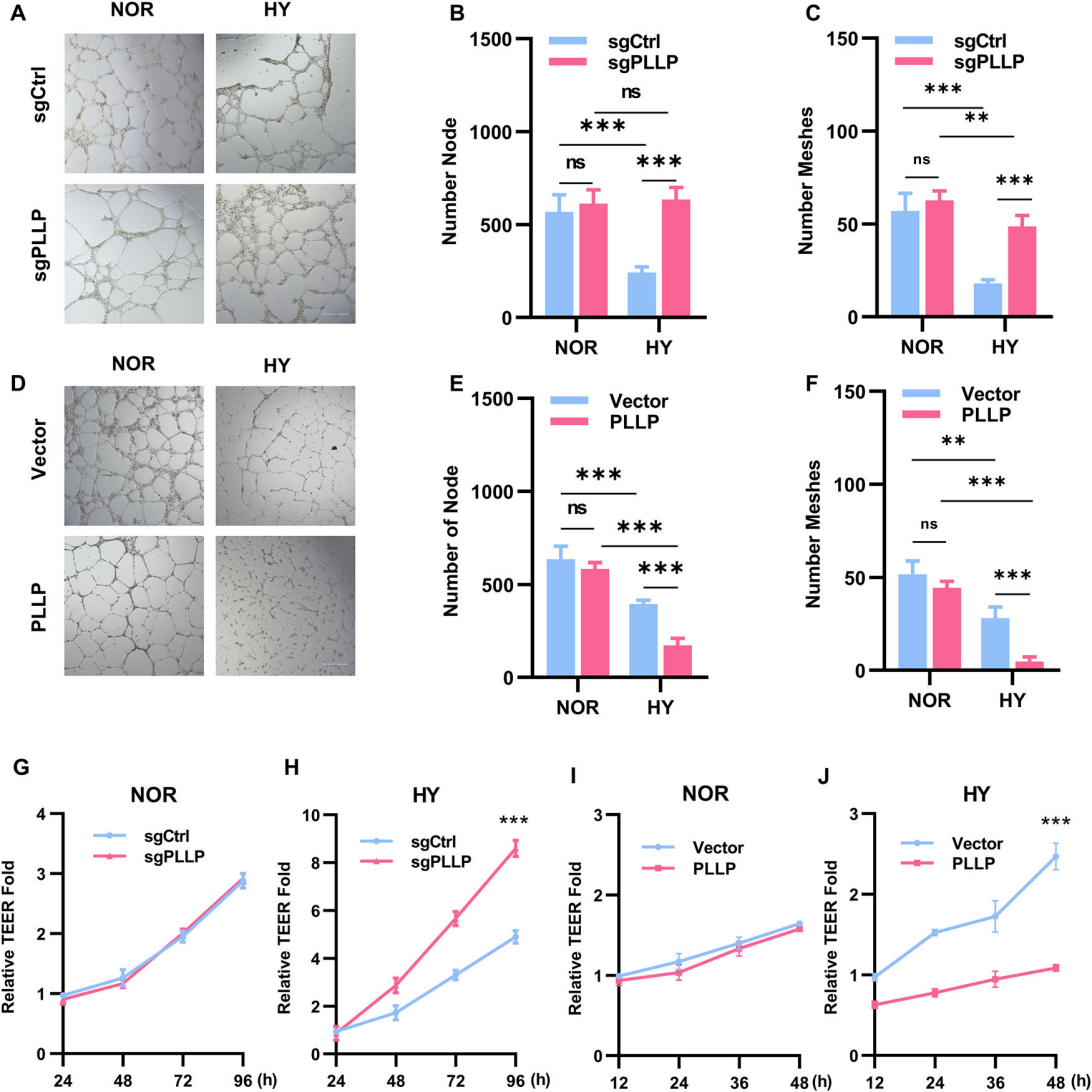
PLLP knockout increased the angiogenic ability of HUVECs in hypoxia. **A**–**C** Angiogenesis assay was used to detect the angiogenic ability of HUVECs with PLLP knocked out after 6 hours of culture under normal and hypoxic conditions. **D**–**F** Angiogenesis assay to detect the angiogenic ability of HUVECs with PLLP overexpressing after 6 hours of culture under normal and hypoxic conditions. **G** Cell resistance meter to detect the membrane electrical resistivity of HUVECs after knocking out PLLP in normoxia. **H** Cell resistance meter to detect the membrane electrical resistivity of HUVECs after knocking out PLLP in hypoxia. **I** Cell resistance meter to detect the membrane electrical resistivity of HUVECs after overexpressing PLLP in normoxia. **J** Cell resistance meter to detect the membrane electrical resistivity of HUVECs after overexpressing PLLP in hypoxia. The results are expressed as the mean ± standard deviation and analyzed using Two-way ANOVA (*n* = 3, *p < 0.05, **p < 0.01, ***p < 0.001, ns=not significant).

**Fig. 5:**
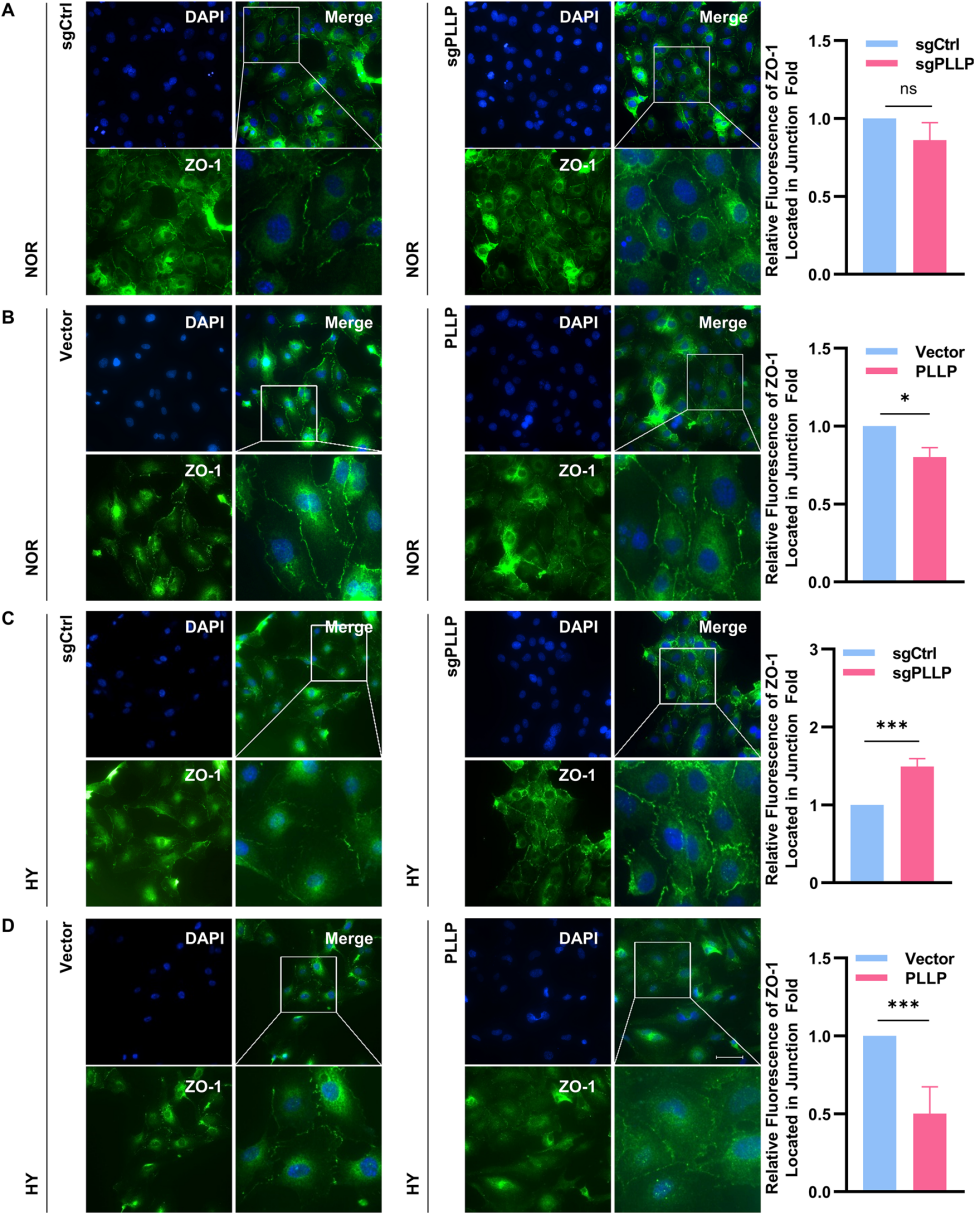
PLLP knockout increased the expression of ZO-1 at the tight junction of HUVECs in hypoxia. **A** Immunostaining on HUVECs after knocking out PLLP in normoxia for 24 hours showing Zona Occludens 1(ZO-1, green). Quantification of ZO-1 expression at intercellular junction with Image J. **B** Immunostaining on HUVECs after knocking out PLLP in hypoxia for 24 hours showing Zona Occludens 1(ZO-1, green). Quantification of ZO-1 expression at intercellular junction with Image J. **C** Immunostaining on HUVECs after overexpressing PLLP in normoxia for 24 hours showing Zona Occludens 1(ZO-1, green). Quantification of ZO-1 expression at intercellular junction with Image J. **D** Immunostaining on HUVECs after overexpressing PLLP in normoxia for 24 hours showing Zona Occludens 1(ZO-1, green). Quantification of ZO-1 expression at intercellular junction with Image J. (Scale bar, 10 μm.) The results are expressed as the mean ± standard deviation. The results are expressed as the mean ± standard deviation and analyzed using a two-tailed Student’s t-test (*n* = 3, *p < 0.05, **p < 0.01, ***p < 0.001, ns=not significant).

HUVECs possess the ability to divide and migrate rapidly in response to angiogenic signals, with their functional state being determined by their angiogenic capacity. In the context of normoxia, the angiogenesis ability of sgPLLP did not exhibit a significant difference compared to sgCtrl (Fig. 4A–C and Fig. S5A–C). Conversely, under hypoxic conditions, the angiogenesis ability of sgPLLP surpassed that of sgCtrl (Fig. 4A–C and Fig. S5A–C), while cells overexpressing PLLP demonstrated weakened angiogenic ability under hypoxia (Fig. 4D–F and Fig. S5D–F). The assessment of HUVECs tight junctions serves as a critical indicator of their growth status. To measure transendothelial electrical resistance (TEER), we compared sgPLLP to sgCtrl under normoxia, observing no disparity in TEER (Fig. 4G). However, under hypoxic conditions, sgPLLP displayed stronger TEER than sgCtrl (Fig. 4H). In addition, PLLP overexpression significantly decreased TEER under hypoxia without affecting TEER under normoxic conditions (Fig. 4I, J). Furthermore, the impact of hypoxia on the communicating junctions of HUVECs was evaluated through the expression analysis of zona occludens 1 (ZO-1). Under hypoxic conditions, a reduction in ZO-1 expression at the cell junction and an increase in the dispersed cytoplasm were noted as compared to normoxic conditions (Fig. 5A, B). Notably, PLLP-depleted cell exhibited significantly higher ZO-1 expression at the cell junction than the control group under hypoxia, while PLLP overexpressing cells showed markedly lower ZO-1 expression at the cell junction than the vector group (Fig. 5C, D). Overall, these findings underscore the critical role of PLLP deficiency in influencing the angiogenic ability and tight junction integrity of HUVECs under hypoxic conditions.

### PLLP deficiency inhibited the killing effect of bevacizumab on HUVECs in hypoxia

**Fig. 6:**
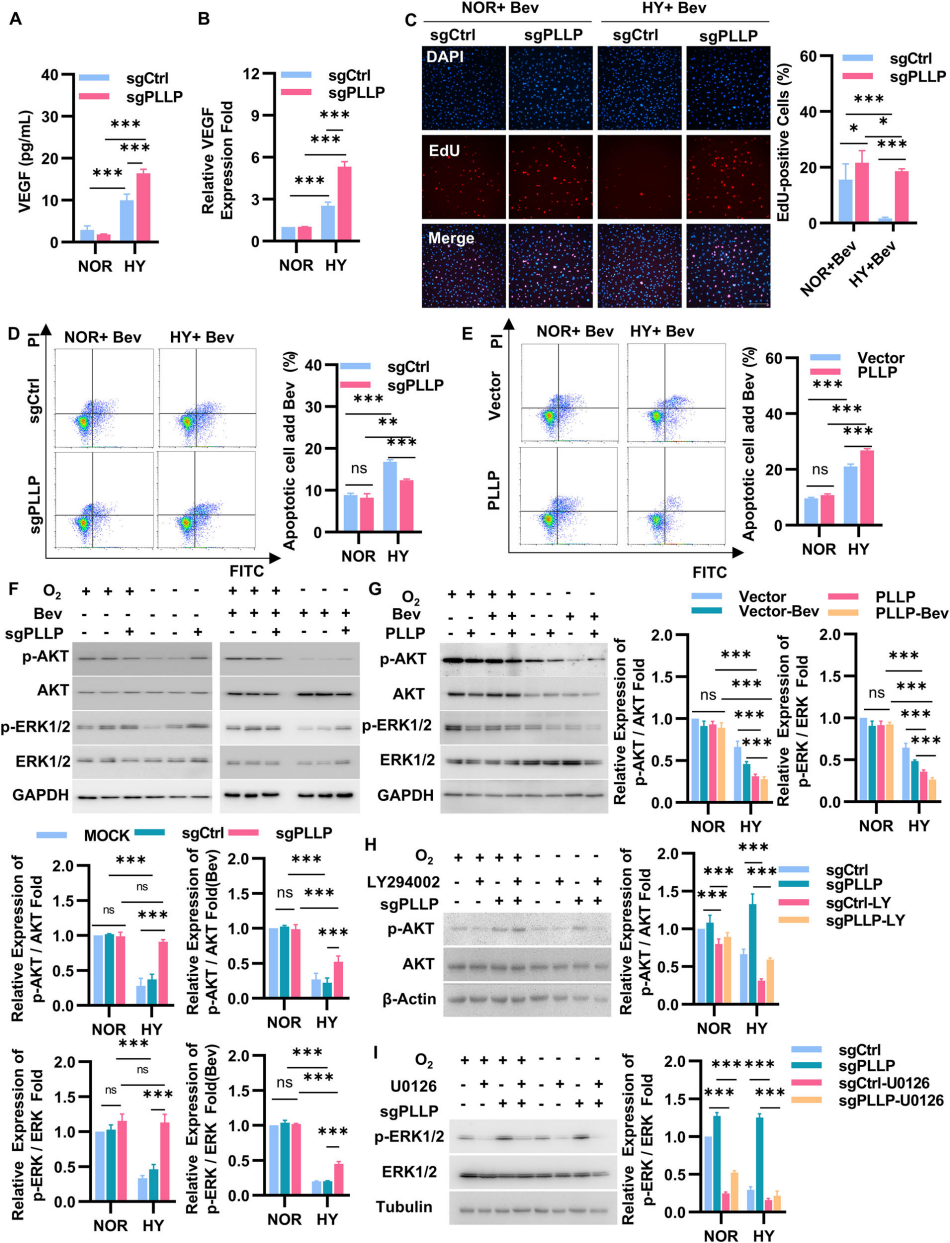
PLLP deficiency inhibited the killing effect of bevacizumab on HUVECs in hypoxia. **A** ELISA assay to detect the VEGF concentration of HUVECs after knocking out PLLP under normal or hypoxic conditions for 24 hours. **B** mRNA expression of VEGF after HUVEC knocking out PLLP under normal or hypoxic conditions for 24 hours. **C** EdU incorporation experiment to detect the proliferation ability of sgPLLP and sgCtrl cells cultured with bevacizumab under normal and hypoxic conditions for 12 hours. Scale bar, 25 μm. **D** Flow cytometry analysis of knockout cells treated with 50 μg/mL bevacizumab for 24 hours in hypoxia or normoxia. **E** Flow cytometry analysis of PLLP overexpressing cells treated with 50 μg/mL bevacizumab for 24 hours in hypoxia or normoxia. **F** Expression of AKT, phospho-AKT, ERK1/2 and phospho-ERK1/2 proteins evaluated by western blot in HUVECs knocking out PLLP treating with bevacizumab or not and cultivated for 24 hours in normoxia or hypoxia. Quantification of western blot with Image J. **G** Expression of AKT, phospho-AKT, ERK1/2 and phospho-ERK1/2 proteins evaluated by western blot in HUVECs overexpressing PLLP treating with bevacizumab or not and cultivated for 24 hours in normoxia or hypoxia. Quantification of western blot with Image J. **H** Expression of AKT and phospho-AKT proteins evaluated by western blot in PLLP knockout HUVECs that treating with LY294002 or not and cultivated for 24 hours in normoxia or hypoxia. Quantification of western blot with Image J. **I** Expression of ERK1/2 and phospho-ERK1/2 proteins evaluated by western blot in PLLP knockout HUVECs that treating with U0126 or not and cultivated for 24 hours in normoxia or hypoxia. Quantification of western blot with Image J. The results are expressed as the mean ± standard deviation and analyzed using Two-way ANOVA (*n* = 3, *p < 0.05, **p < 0.01, ***p < 0.001, ns not significant).

Following the knockout of PLLP in cells, an interesting observation was made regarding the enhanced expression of VEGF under hypoxic conditions compared to the control group sgCtrl (Fig. 6A, B). Bevacizumab serves as a VEGF inhibitor, impacting tumor cell survival by specifically binding to VEGF [43]. As a result, our data prompted an investigation into whether PLLP ‘s presence under hypoxic conditions influenced the inhibitory impact of bevacizumab on the survival of HUVECs. Treatment of HUVECs with bevacizumab under normoxic or hypoxic conditions was carried out. It was observed that under normoxic conditions, the proliferation rate of sgPLLP significantly increased in comparision to sgCtrl (Fig. 6C). Notably, under hypoxic conditions, sgCtrl exhibited a substantial reduction in proliferation compared to normoxic conditions, sgPLLP was only marginally affected in terms of proliferation ability (Fig. 6C). Subsequent cell apoptosis assays verified the aforementioned findings. Specifically, under normoxic conditions, the sgPLLP demonstrated lower apoptosis rates than the sgCtrl, while under hypoxic conditions, the apoptosis rate of sgPLLP is lower than that of sgCtrl (Fig. 6D). The apoptosis rate of sgCtrl increases significantly under hypoxic conditions compared to normoxic conditions while the apoptosis rate of sgPLLP only shows a slight increase under hypoxic conditions compared to normoxic conditions (Fig. 6D). Importantly, in hypoxic conditions, cells overexpressing PLLP exhibited higher apoptosis rates than the vector, with no significant disparity in apoptosis rates detected under normoxic conditions (Fig. 6E). Collectively, these findings suggest that PLLP knockout diminishes the cytotoxic effect of bevacizumab on HUVECs under hypoxic conditions.

Our research initially elucidated the significant impact of PLLP knockout on enhancing the survival of HUVECs during hypoxic condition. To further explore the specific protein within signaling pathways affected by PLLP, we conducted western blotting analysis to evaluate the expression levels of key signaling pathway proteins in HUVECs. Western blot experiments were performed on various cell groups including HUVECs, sgCtrl, sgPLLP, PLLP overexpressing HUVECs, and all groups subjected to bevacizumab treatment. The phosphorylated AKT and phosphorylated ERK pathway is an intracellular signal transduction pathway that promotes metabolism, proliferation, cell survival, growth, and angiogenesis in response to extracellular signals [44–46]. The protein expression levels of phosphorylated AKT and ERK were assessed, followed by an evaluation of the total protein levels of AKT and ERK. Subsequently, we calculated the ratio of phosphorylated protein to total protein in order to quantify the phosphorylation levels. In the normoxic setting, there was no discernible difference in the phosphorylation levels between AKT and ERK1/2 in sgCtrl and sgPLLP groups. However, under hypoxic conditions, the phosphorylation of AKT and ERK1/2 was markedly higher in sgPLLP cells compared to sgCtrl cells. Interestingly, treatment of sgCtrl cells with bevacizumab resulted in reduced phosphorylation levels of AKT and ERK1/2 in normoxia, whereas the sgPLLP group exhibited upregulation. In normoxic conditions, bevacizumab treatment reduced AKT and ERK1/2 phosphorylation in both control and PLLP-overexpressing cells, with the reduction being more pronounced in the latter (Fig. 6G). Conversely, under hypoxic conditions, sgCtrl cells showed decreased AKT and ERK1/2 phosphorylation compared to normoxia, whereas sgPLLP cells were less affected by hypoxia-induced changes (Fig. 6F). Notably, PLLP-overexpressing cells exhibited significantly lower AKT and ERK1/2 phosphorylation under hypoxia compared to vector controls, with the lowest phosphorylation levels observed in PLLP-overexpressing cells treated with bevacizumab (Fig. 6G). In contrast to these in vitro findings, in vivo analysis using cerebral ischemia and hindlimb ischemia models revealed increased AKT and ERK expression (Fig. S6A and S6B). To investigate whether PLLP directly modulates AKT/ERK signaling, we treated cells with the ERK1/2 inhibitor U0126 and the AKT inhibitor LY294002. As shown in Figure S6C, D, inhibitor treatment significantly reduced AKT and ERK1/2 phosphorylation in PLLP-overexpressing cells. Conversely, in PLLP knockout cells under hypoxia, the inhibitors failed to suppress AKT and ERK1/2 phosphorylation (Fig. 6H, I), suggesting that PLLP knockout directly impacts the AKT and ERK1/2 signaling pathways. Collectively, these results indicate that PLLP knockout enhances HUVEC survival under hypoxic conditions, and that disrupting PLLP function attenuates bevacizumab’s inhibitory effects on HUVEC survival during hypoxia by increasing AKT and ERK1/2 phosphorylation.

## Discussion

Adequate oxygen concentration is crucial for maintaining homeostasis in biological tissues; hypoxia, characterized by diminished oxygen availability, leads to reduced cell growth and vitality [47]. In the current research, HIF, GLUT and CA IX are currently the most studied factors related to hypoxia, and VEGF is a star molecule closely related to endothelial cells [48–51]. Since its first isolation of PLLP from the plasma membrane of bovine kidney cells in 1981, PLLP has been the subject of studies primarily focused on its roles in kidney and membrane cell [52, 53]. The human PLLP gene, spanning approximately 28.5 kb, encodes small molecular weight proteolipid protein. Notably, PLLP features a MARVEL domain, shared with MAL-related proteins associated with vesicular transport and membrane interactions [54], suggesting its role in vesicular transport and tight junction-associated processes [55]. PLLP controls Crb3(Crumbs3) levels by modulating apical endocytosis and induces precise regulation of Notch activity in Notch-receptor expressing cells [56]. Additionally, PLLP is co-localizes with Occludin and Claudin-4 in the tight junctions on basolateral plasma membranes of MDCK cells [57]. In this study, we employed CRISPR/Cas9 screening to investigate the relationship between PLLP and hypoxia, aiming to identify novel avenues for hypoxia research. Our findings indicate that while PLLP knockout does not affect the growth of HUVECs under normoxic conditions, it confers resistance to the detrimental effects of hypoxia on HUVEC activity, cellular metabolism, cell migration, and angiogenesis. Notably, we did not observe any impact of PLLP on the expression levels of key hypoxia factors such as HIF-1α.

In our study, we observed that under hypoxic conditions, knockout of PLLP in HUVECs led to increased cell migration and angiogenesis. This was accompanied by elevated ZO-1 expression and its enhanced localization at the cell membrane, resulting in increased cell permeability. Mechanistically, PLLP knockout under hypoxia upregulated the phosphorylation levels of AKT and ERK1/2, while PLLP overexpression inhibited this phosphorylation, suggesting that PLLP potentially regulates cell migration and angiogenesis through these signaling pathways. Specifically, activation of AKT, typically triggered by growth factors, cytokines, or other cellular stimuli, involves phosphorylation at its phosphorylation site, leading to the activation of downstream signaling pathways and ultimately increased cell migration [58]. Similarly, the binding of signaling factors to the phosphorylation site activates ERK phosphorylation, influencing angiogenesis [46]. ZO-1, known to regulate intestinal epithelial cells through interactions involving tight junction proteins and cell actin, modulates cell permeability. Stimulation of tight junctions can decrease ZO-1 expression and alter its distribution, changing the permeability to ions and small molecules, thus affecting the potential difference across the cell membrane and overall cell permeability [59, 60]. ZO-1 affects the migration ability of endothelial cells by regulating the remodeling of the actin cytoskeleton, and helps endothelial cells form tubular structures by coordinating the dynamic assembly of tight junctions and adhesion junctions. Furthermore, ZO-1 influences endothelial cell migration by regulating actin cytoskeleton remodeling and facilitates the formation of tubular structures by coordinating the dynamic assembly of tight and adhesion junctions. Supporting these observations, our findings also demonstrated that overexpressing PLLP markedly reduced the survival and angiogenic capacities of HUVECs under hypoxia, further indicating a protective role of PLLP against tumor cell transformation in a hypoxic microenvironment.

Bevacizumab, the first clinically used anti-angiogenic drug, is a humanized monoclonal antibody that binds to VEGF-A, preventing its interaction of VEGF-A with VEGFR and thereby inhibiting the activation of the VEGF signaling pathway that promotes neovascularization [61]. Previous studies have shown that bevacizumab not only inhibits vascular growth and induces the regression of newly formed blood vessels but also normalizes the vasculature to enhance the delivery of cytotoxic chemotherapy [61]. This study confirmed that PLLP influences the inhibitory effect of bevacizumab in hypoxia. In addition, bevacizumab demonstrated effectiveness inhibiting the phosphorylation of AKT and ERK1/2 in HUVECs that overexpress PLLP. Our study preliminary explorating the potential of a synergistic relationship between bevacizumab and PLLP. This suggests the possibility of developing PLLP or PLLP activator as protein drugs in the future, to be used in combination with bevacizumab for enhanced treatment efficacy.

## Materials and methods

### Animal models

All animal procedures were conducted in accordance with the ethical guidelines of the Beijing Institute of Biotechnology and the recommendations of the Beijing Experimental Animal Regulation Board (approval no. IACUC-SWGCYJS-2025-025). Male and female C57BL/6 mice, aged 4-8 weeks, were obtained from Beijing Vital River Laboratory Animal Technology Co., Ltd. (China) and used for all experiments. Randomization or blinding was not performed. Prior to experimentation, all animals were confirmed to be healthy and without prior experimental or drug treatment history. Mice were housed in a pathogen-free facility under a 12-hour light/dark cycle (6:00 AM to 6:00 PM), with ad libitum access to food and water. At the conclusion of the experiments, animals were euthanized via intravenous injection of pentobarbital sodium (150 mg/kg) for tissue collection.

### Cerebral ischemic model

All surgical procedures were performed under aseptic conditions by an experienced mouse surgeon. Throughout the surgery, body temperature was maintained at 36.5 ± 0.5 °C using warm water pads and a heating lamp. Anesthesia was induced with 1% Nembutal, and a 2 cm midline incision was made in the anterior neck. The right common carotid artery was then ligated using 7-0 silk sutures. After 45 min of occlusion, the suture was carefully removed to allow for reperfusion. Finally, the incision was closed with 4-0 surgical sutures. *n* = 6.

### Hindlimb ischemia model

Following induction of anesthesia with 1% Nembutal in 8-week-old C57BL/6 wild-type mice, the animals were placed on a circulating heated water pad to maintain body temperature. A 1 cm incision was then made in the left groin region. Utilizing a Nikon SMZ800 microscope, the neurovascular pedicle was visualized, and the femoral nerve was carefully dissected. The femoral vein, located medially, was then separated from the laterally positioned femoral artery, taking care to spare both the vein and nerve. Subsequently, the left common femoral artery was electrocoagulated proximal to the bifurcation of the superficial and deep femoral arteries. Finally, the surgical site was inspected for any residual bleeding to ensure complete occlusion. *n* = 6.

### Cell culture and environment stimulation

The Immortalizated HUVECs and Primary HUVECs (Hefei Wanwu Biotechnology Co., LTD) were routinely cultured in Endothelial Cell Medium (ECM, ScienCell^TM^, USA), which was supplemented with 5% fetal bovine serum, 100 units/mL of penicillin, 100 mg/mL of streptomycin and 1% ECGF (Endothelial Cell Growth Factor) [42, 62]. In normoxic experiments or conventional culture, cells were incubated in a humidified chamber with 5% CO_2_ and 21% O_2_ at 37 °C. In hypoxic experiment, HUVECs were incubated in a humidified chamber with 5% CO_2_ and 1% O_2_ at 37 °C. The HUVECs were authenticated STR profiling and tested for mycoplasma contamination.

### PLLP knockdown of primary HUVECs

Human PLLP expression was inhibited using 50 nM siRNA against PLLP (TsingkeBiotechnologyCo. Ltd.; 5’-CACUGGUGUUAAUGAUCUU- ‘3) denoted as siPLLP in the text. In parallel, a Negative Control siRNA (TsingkeBiotechnologyCo. Ltd., China) was used, which are denoted as siNC. For experiments, primary HUVEC were transfected with control or PLLP siRNA (50 nM) using Lipofectamine® 2000 Reagent (Thermo Fisher Scientific, USA). After 6 h, the media was replaced and cells were collected after 48 h and RNA extracted.

### Construction of CRISPR/Cas9 cells and sgRNA candidate selection

HUVECs were inoculated in 6-well plate and cultured for 12 hours. When the confluence reached at 70%, the cells were infected with Cas9 lentivirus and incubated in a humidified chamber with 5% CO_2_ at 37 °C. The Cas9 constructed by lentivirus was purchased from Genechem Co. Ltd (Shanghai, China). Infecting with lentivirus after 12 h, monoclone cells stably expressing Cas9 were screened with puromycin, which is the negative control.

Add 2 × 10^6^ negative control cells were inoculated in a 10 mm cell culture dish and infected with GeCKO v2 library lentivirus. Lentivirus constructing of GeCKO v2 library was purchased from ZhangLab. The GeCKO v2 library consists of specific sgRNA sequences used for gene knockout in human or mouse genomes. Cells were incubated in a humidified chamber with 5% CO_2_ and 1% O_2_ at 37 °C, so as to screen out monoclonal cells that survived under hypoxia. Validated by PCR and high-throughput sequencing after screening monoclone cells.

### Construction of PLLP knockout or overexpression cells

PLLP-gRNA was constructed on lentiCRISPR-v2 vector, and lentiCRISPR-PLLP-gRNA was transfected into HUVECs alone in the form of lentivirus. HUVECs were inoculated in 6-well plate and cultured for 12 h. When the confluence reached at 70%, the cells were infected with lentiCRISPR-PLLP-gRNA and incubated in a humidified chamber with 5% CO_2_ at 37 °C. Infecting with lentivirus after 12 h, monoclone cells were screened with puromycin in 5% CO_2_ and 1% O_2_ at 37 °C, and this condition was used to exclude the mixed clones in the previous library screening [41]. Lentiviruses overexpressing PLLP were purchased from GenePharma (Shanghai, China). HUVECs were inoculated in 6-well plate and cultured for 12 h. When the confluence reached at 70%, the cells were infected with lentiviruses and incubated in a humidified chamber with 5% CO_2_ at 37 °C. Infecting with lentivirus after 12 h, cells were screened with puromycin.

### Genome extraction and PCR detection

Genome was isolated from cells using the DNeasy Blood & Tissue Kit (QIAGEN, USA). Difference between genome expressions was presented as normalization of the GAPDH or level. The primers sequence is shown in Supplemental Table 1.

### Quantitative real-time polymerase chain reaction(qRT-PCR)

Total RNA was isolated from cells using the RNeasy Mini Kit 50 (QIAGEN, USA). Complementary DNA (cDNA) was conducted using High-Capacity cDNA Reverse Transcription Kit (Thermo Fisher, USA) with 1 μg of total RNA. The primers sequence is shown in Supplemental Table 1. Each measurement is the average of three independent experiments.

### Western blot

After blocking 1 hour at room temperature, membranes were incubated with the primary antibodies 1 h at room temperature. After washing away the primary antibody, the membranes were then incubated with HRP-conjugated secondary antibodies 1 h at room temperature. After washing, added Western Lightning Plus ECL (PerkinElmer, USA), and protein bands were visualized using ChemiDocTM Imaging System (BIO-RAD, USA). Image J was used to analyze the grayscale values. Each measurement is the average of three independent experiments.

### Antibodies

P-AKT Rabbit mAb (#4060,1:2000), AKT Rabbit mAb (#4691,1:1000), P-p44/42 MAPK (ERK1/2) Rabbit mAb (#4370,1:2000), p44/42 MAPK (ERK1/2) Rabbit mAb (#4695,1:1000) and GAPDH Rabbit mAb (#2118,1:3000) were bought from Cell Signaling Technology (CST, USA). Rabbit pAb to PLLP antibodies (ab236668,1:1000) and Rabbit pAb to ZO-1 antibodies (ab216880,1:1000) were bought from Abcam (Abcam, USA).CD31 Rabbit PolyAb(11265-1-AP,1:500) were bought from Proteintech (Proteintech Group, Inc, USA). Goat Anti-Rabbit IgG and Goat Anti-Mouse IgG were bought from ZSGB-BIO (Beijing, China,1:3000).

### Immunofluorescent staining

For immunofluorescence, cells were seeded at a density of 2 × 10^5^ per 24-well, then washed with phosphate-buffered saline (PBS) and fixed with 4% paraformaldehyde (15 min, 37 °C). The cells were permeabilized with 0.3% Triton X-100 for 10 min and blocked with 10% bovine serum for 1 h and stained with primary antibodies at 4 °C overnight. After washing with PBS, cells were stained with Alexa-488-conjugated secondary antibodies (50 min, room temperature) and with DAPI. Digital pictures were captured using a fluorescence microscope. Each measurement is the average of three independent experiments. Image J was used to analyze the fluorescence values.

### Immunohistochemical staining

Immunohistochemical staining was performed on paraffin-embedded tissue sections. Initially, sections with a thickness of 5 μm were deparaffinized, rehydrated, and subjected to antigen retrieval by heating at 100 °C in 0.01 M citrate buffer. Endogenous peroxidase activity was then quenched by incubation with 3% hydrogen peroxide for 20 minu. To block non-specific binding, sections were treated with normal goat serum, followed by overnight incubation at 4 °C with the primary antibodies. After rinsing with PBS, the sections were incubated with goat anti-rabbit IgG for 1 hour. Finally, all sections were counterstained with hematoxylin, dehydrated, and sealed for microscopic examination. These tissue sections were obtained from our previous study.

### Cell viability

The viability of HUVECs was tested by Cell Titer-Glo® Luminescent Cell Viability Assay Kit (Promega, USA). Concretely, the cells were plated into 96-well plates and cultured for 0, 6, 12, 18, 24 h in normoxia or hypoxia. Then, each well was treated with 100 μL of Cell Titer-Glo^®^Reagent (Promega, USA) for 10 minutes at room temperature. The absorbance at full wavelength was recorded by a microplate spectrophotometer. The change of luminescence value indicated the change of cell vitality. Each detection value was the average of three independent experiments.

### Cell apoptosis

The cells were seeded into 12-well plate at a density of 2 × 10^5^ cells/well. In inhibitor experiment, Bevacizumab (APExBIO, USA) was added into the wells at the concentration of 25 mg/mL. After incubation at 37 °C for 24 or 48 h the cells were collected for apoptosis analysis using an Annexin V-FITC/PI apoptosis assay kit (Beyotime Biotechnology, China). Briefly, cells were stained with Annexin V-FITC and PI for 10 min at 4 °C in the dark. Then the cells were analyzed immediately by Attune NxT flow cytometer (Thermo Scientific, USA) and the primary data was analyzed with Flowjo software (Tree Star, USA.). Each measurement was the average of three independent experiments.

### Wound-healing

Cells grown in 6 well plates were artificially injured by scratching across the plate with 200 μL pipet tips. The wound areas were photographed at the indicated time points and measured using ImageJ software (NIH, Bethesda, MD, USA). Wound closure percentages were calculated using the following formula: (1 - [current wound size/initial wound size]) ×100%. Wound closure was analyzed using Image J. Each measurement is the average of three independent experiments.

### Cell migration and invasion

The cells were serum-starved overnight, seeded at a density of 2 × 10^4^ cells/well, respectively, in the upper chambers of a 24-well transwell with 8 μm pore-size polycarbonate membrane filter (CORNING, USA) coated with or without Matrigel (CORNING, USA) for the invasion and migration assays. ECM Medium containing 5% FBS was added to the lower chambers as a chemoattractant. After incubation at 37 °C for 24 hours with invasion or 12 h with migration, the non-invading cells were gently removed with a cotton swab, while the invasive cells located on the lower surface of upper chambers were fixed in 95% ethanol for 30 min and stained with crystal violet for 30 minutes respectively. The invasive cells were counted by an inverted microscope from five random fields and experiment was repeated three times. The quantity of cell was counted using ImageJ software.

### Cell proliferation

The proliferation activity of cells was tested by BeyoClick™ EdU Cell Proliferation Kit (Beyotime Biotechnology, China). Concretely, the cells were plated into 24-well plates and cultured for 12 or 24 hours in hypoxia or conventionally cultured. Then, each well was treated by BeyoClick™ EdU Cell Proliferation Kit according to protocol. The quantity of cell was counted using ImageJ software. The cells were counted by microscope from random fields and experiment was repeated three times.

### Cell viability

The viability of HUVECs was tested by Cell Titer-Glo® Luminescent Cell Viability Assay Kit (Promega, USA). Concretely, the cells were plated into 96-well plates and cultured for 0, 6, 12, 18, 24 hours in normoxia or hypoxia. Then, each well was treated with 100 μL of Cell Titer-Glo^®^Reagent (Promega, USA) for 10 minutes at room temperature. The absorbance at full wavelength was recorded by a microplate spectrophotometer. The change of luminescence value indicated the change of cell vitality. Each detection value was the average of three independent experiments.

### Oxygen consumption measurements

Bioenergetics changes resulting from hypoxia in HUVECs were investigated by measuring the OCR using a Seahorse XFe24 Analyzer (Agilent, USA). Cells were plated in Seahorse XF24 plates (Agilent, USA) at 1.5 × 10^4^ cells per well and were treated in normoxic and hypoxic environment for 24 h respectively after the cells adhered. The cell culture medium was then removed and replaced with Seahorse XF DMEM Medium (Agilent, USA, supplemented with 10 mM glucose,1 mM pyruvate and 2 mM glutamine) and treated for 1 hour at 37 °C without CO_2_. OCR analysis was performed with the sequential injections of oligomycin (1.5 μM), FCCP (1 μM) and rotenone plus antimycin A mix (0.5 μM) where indicated following the Seahorse XFe24 Analyzer program (XF Cell Mito Stress Test).

### Enzyme linked immunosorbent assay (ELISA)

After 24 hours of cultivation in normoxia or hypoxia, supernatant fluid of HUVECs cells were collected from each group, and then centrifuged at 12,000 × g for 5 min. Freeze the sample at −80 °C or immediately analyze blank cells, control group cells, and PLLP knockout cells using a human ELISA kit (Beyotime Biotechnology, China). We followed the manufacturer’s instructions for all procedures and measured the absorbance at 450 nm using an enzyme-linked immunosorbent assay (Bio Rad, USA). Each measurement is the average of at least three independent experiments.

### Angiogenesis assay in Matrigel

The formation of capillary networks was determined in Matrigel by conducting in vitro formation measurements. Dilute Matrigel (CORNING, USA) in a 1:1 ratio using serum-free and growth factor free medium, and 9 × 10^4^ cells were inoculated onto a 24-well plate coated with diluted Matrigel. After 5 h of cultivation, the formation of the test tube was examined under a microscope (Nikon, Japan), and the branching points and tube length were quantified using Image J software. Each measurement is the average of three independent experiments.

### Cell trans endothelial electrical resistance (TEER) assay

The cells were seeded into 4 μm pore-size polycarbonate membrane filter (CORNING, USA) for co-cultured at a density of 2 × 10^4^ cells/well, and the cells were cultured in the same medium in the upper chamber and the lower chamber. The cells TEER was measured by Millicell®ERS-2 (Millipore, USA) at 24, 48, 72 and 96 h or 12, 24, 36 and 48 hours. The measured value was the resistance value that called R. Resistance per unit area were calculated using the following formula: R per unit area=R/Effective membrane area. Each measurement was the average of three independent experiments. The 24 h measurement value of the negative control group was used as the standard for normalization.

### Statistical analysis

To ensure the reproducibility of findings, all results presented represent a minimum of at least three biological replicates independent experiments. Randomization or blinding was not performed. No statistical method was used for predetermined sample size. Data are expressed as the mean ± standard deviation (SD). Statistical significance was determined using GraphPad Prism 8 software. For comparisons between two groups with normally distributed data and equal variances were analyzed using a two-tailed Student’s t-test. Two-way ANOVA was used to assess differences among groups considering multiple factors. As indicated in the figure legends, either a representative experiment or pool is shown, and the number of repetitions for each experiment and the number of experimental repeats are indicated. A *p*-value of less than 0.05 was considered statistically significant, with significance indicated as follows: *p < 0.05, **p < 0.01, ***p < 0.001, and ns = not significant.

## Supplementary information

Original Images-WB

Additional Information

Supplement Table 1 Primers for PCR detection

Supplemental Figure Legend

Supplemental figure1

Supplemental figure2

Supplemental figure3

Supplemental figure4

Supplemental figure5

Supplemental figure6

## Data Availability

The data generated during the current study are available from the corresponding author upon reasonable request.
