# Supplementary figures and images for: Plasmolipin deficiency is essential for HUVECs survival under hypoxic conditions
Source: Cell Death Discov. 2025 May 17;11:239. doi: 10.1038/s41420-025-02526-5 (PMC12084367; doi:10.1038/s41420-025-02526-5)

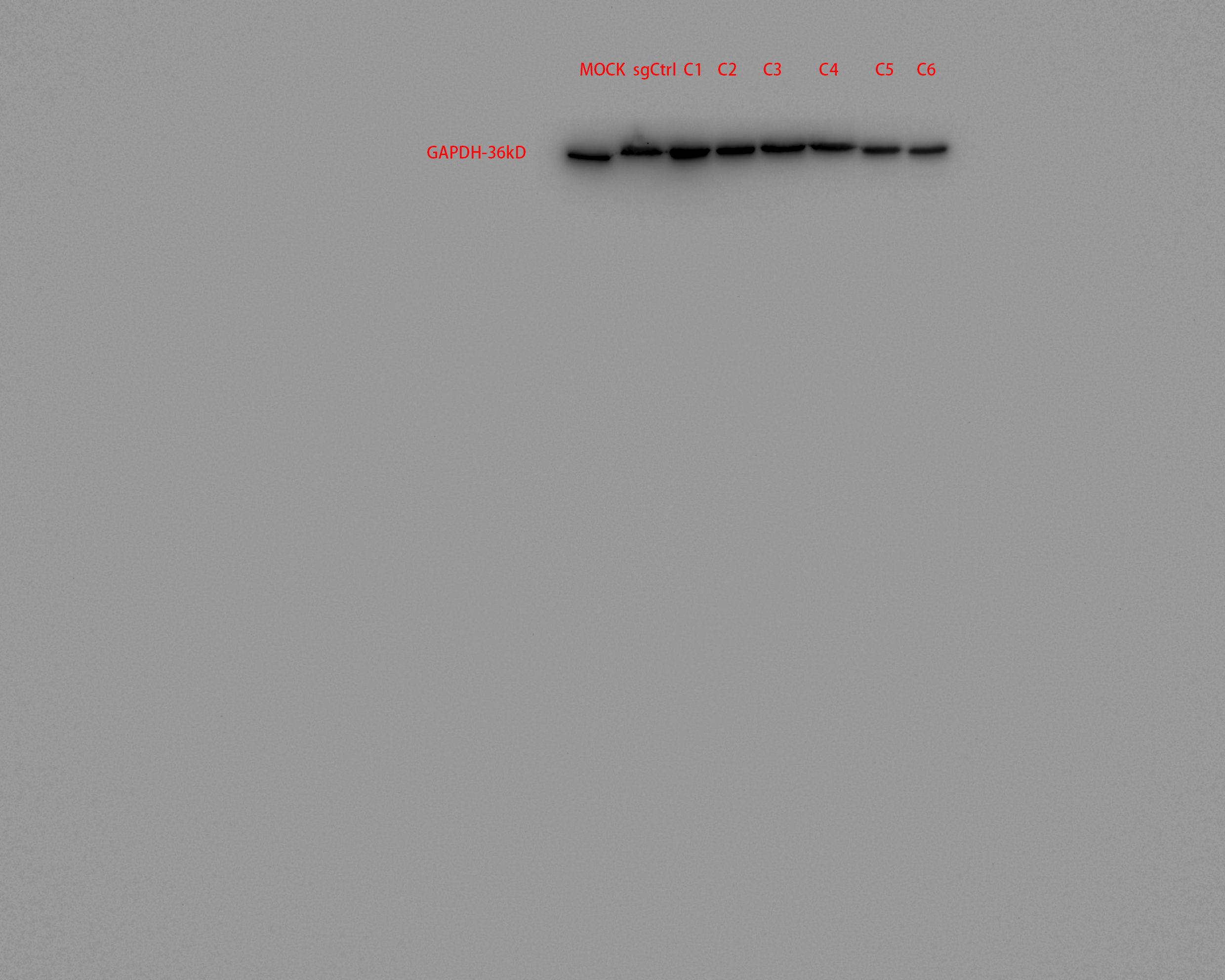

Supplement: Supplementary file 1 — Original Images-WB [file 41420_2025_2526_MOESM1_ESM.zip › Original Images-WB/Fig1H-GAPDH.tif]

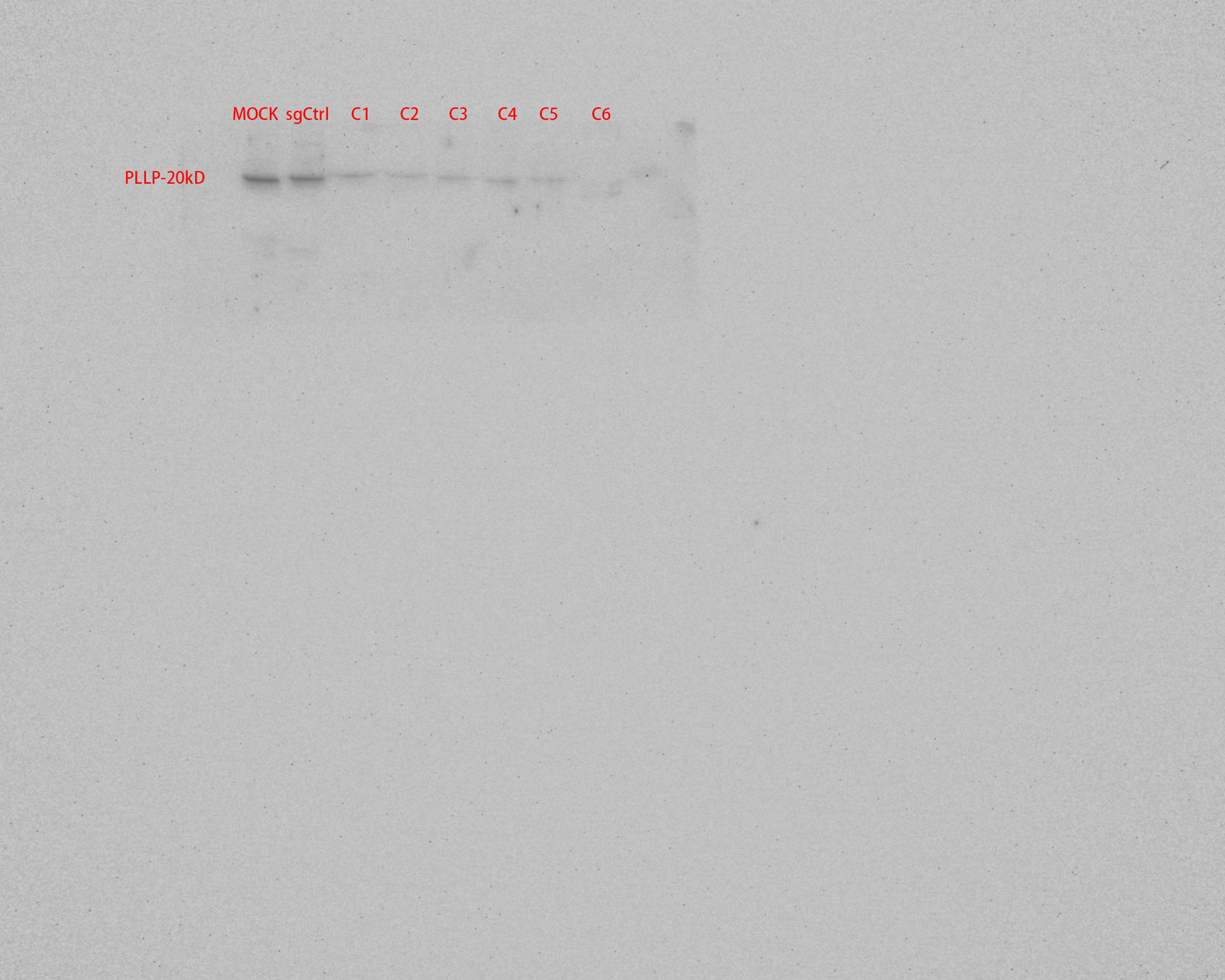

Supplement: Supplementary file 1 — Original Images-WB [file 41420_2025_2526_MOESM1_ESM.zip › Original Images-WB/Fig1H-PLLP.tif]

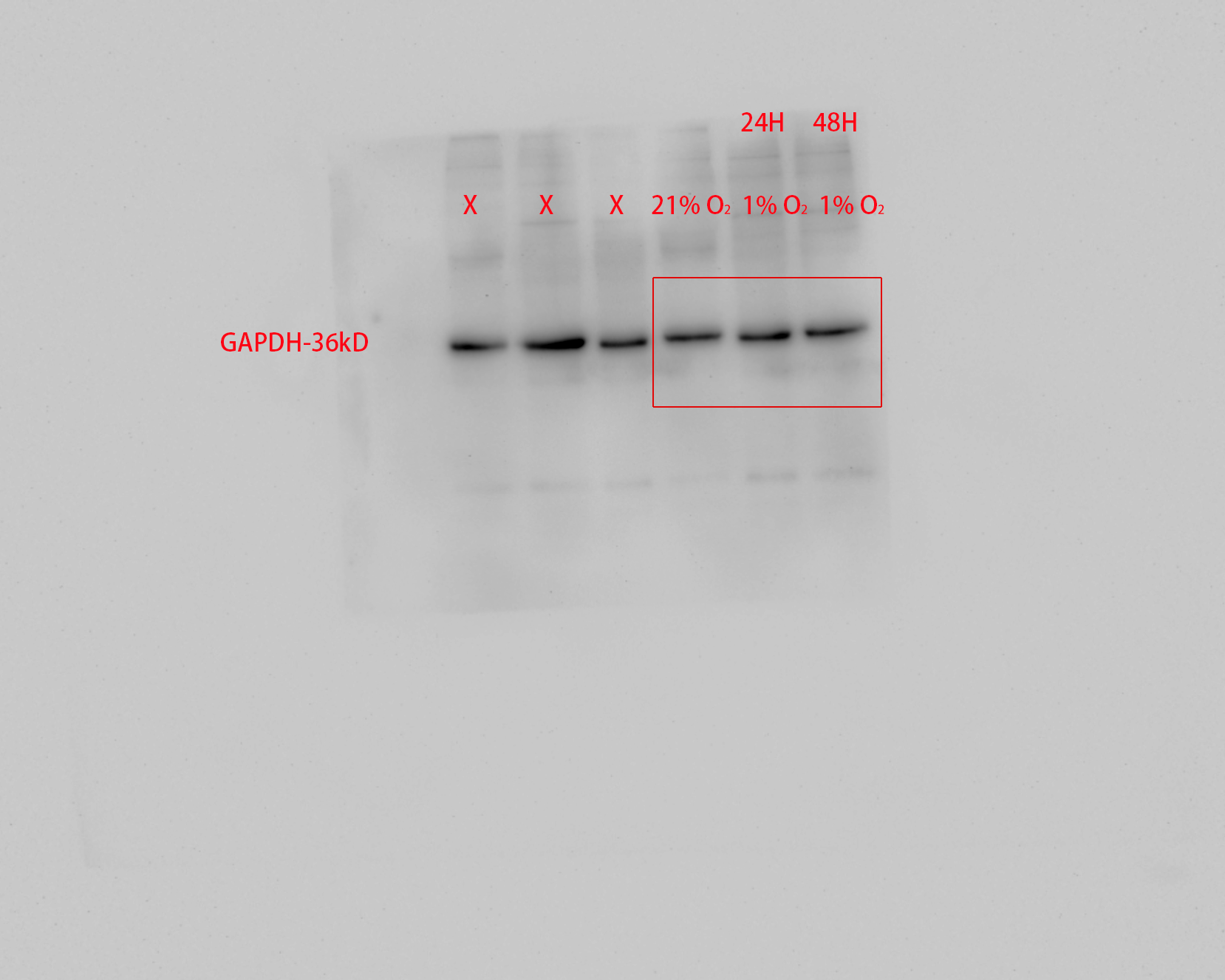

Supplement: Supplementary file 1 — Original Images-WB [file 41420_2025_2526_MOESM1_ESM.zip › Original Images-WB/Fig1J-GAPDH.tif]

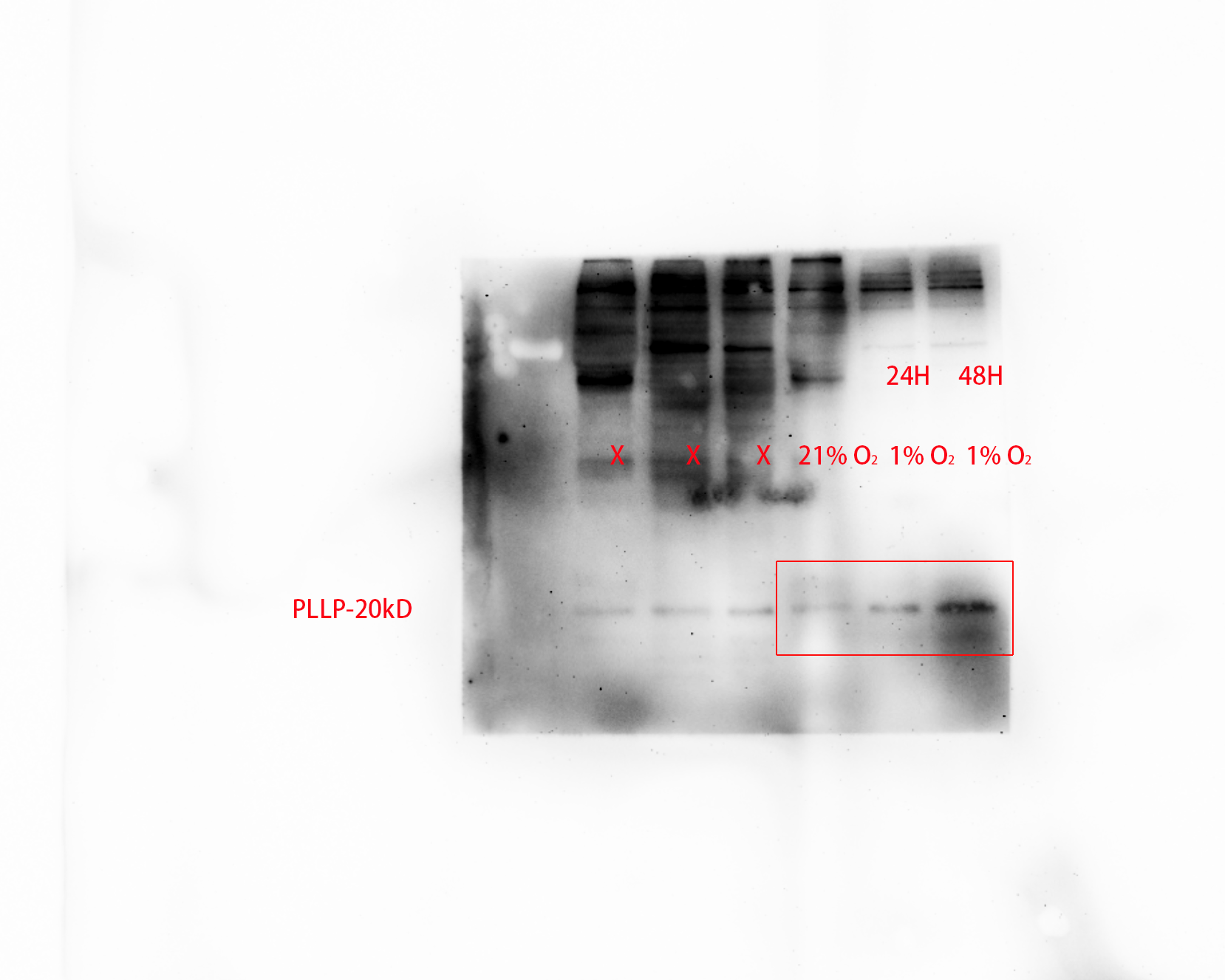

Supplement: Supplementary file 1 — Original Images-WB [file 41420_2025_2526_MOESM1_ESM.zip › Original Images-WB/Fig1J-PLLP.tif]

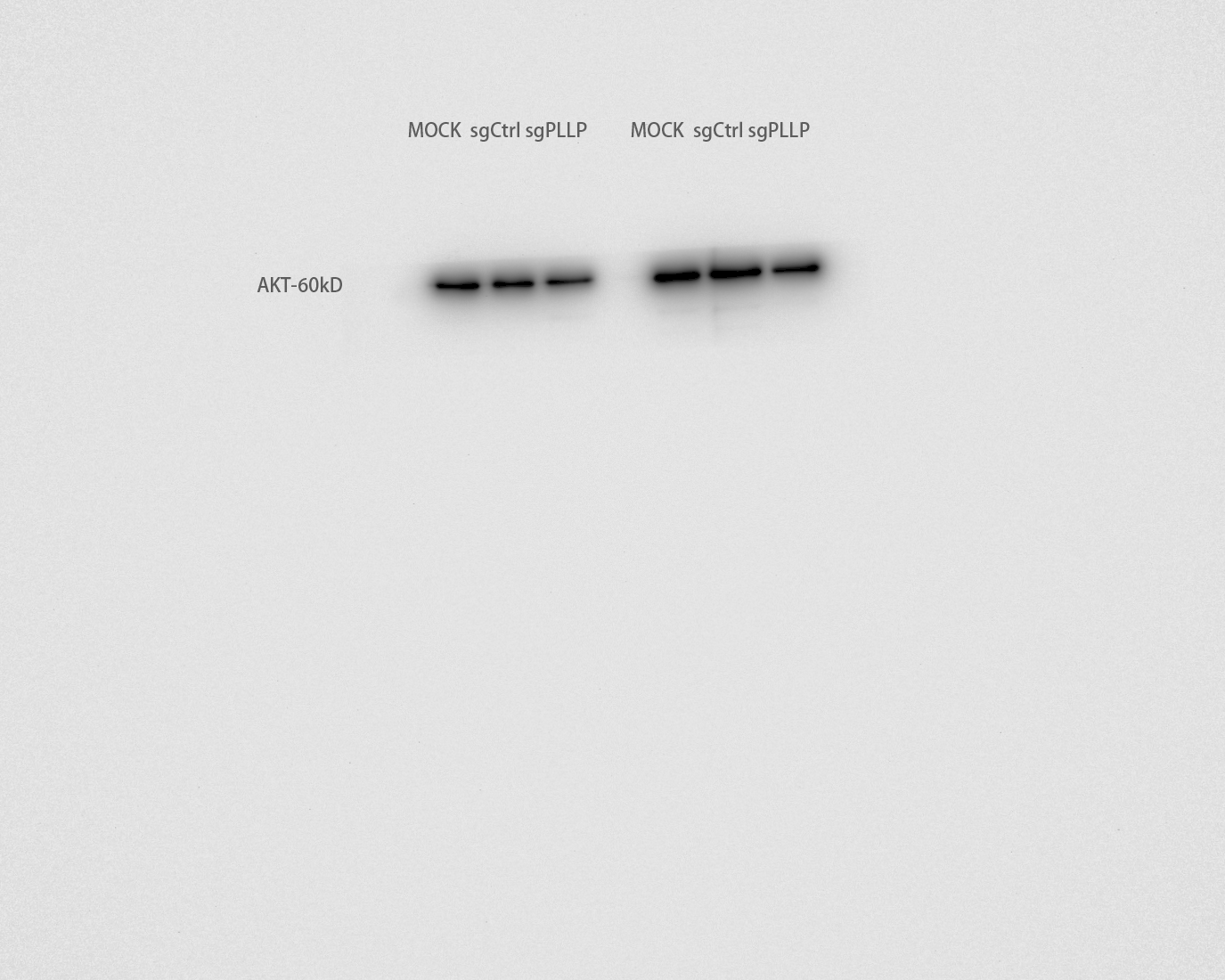

Supplement: Supplementary file 1 — Original Images-WB [file 41420_2025_2526_MOESM1_ESM.zip › Original Images-WB/Fig6F-AKT-bev.tif]

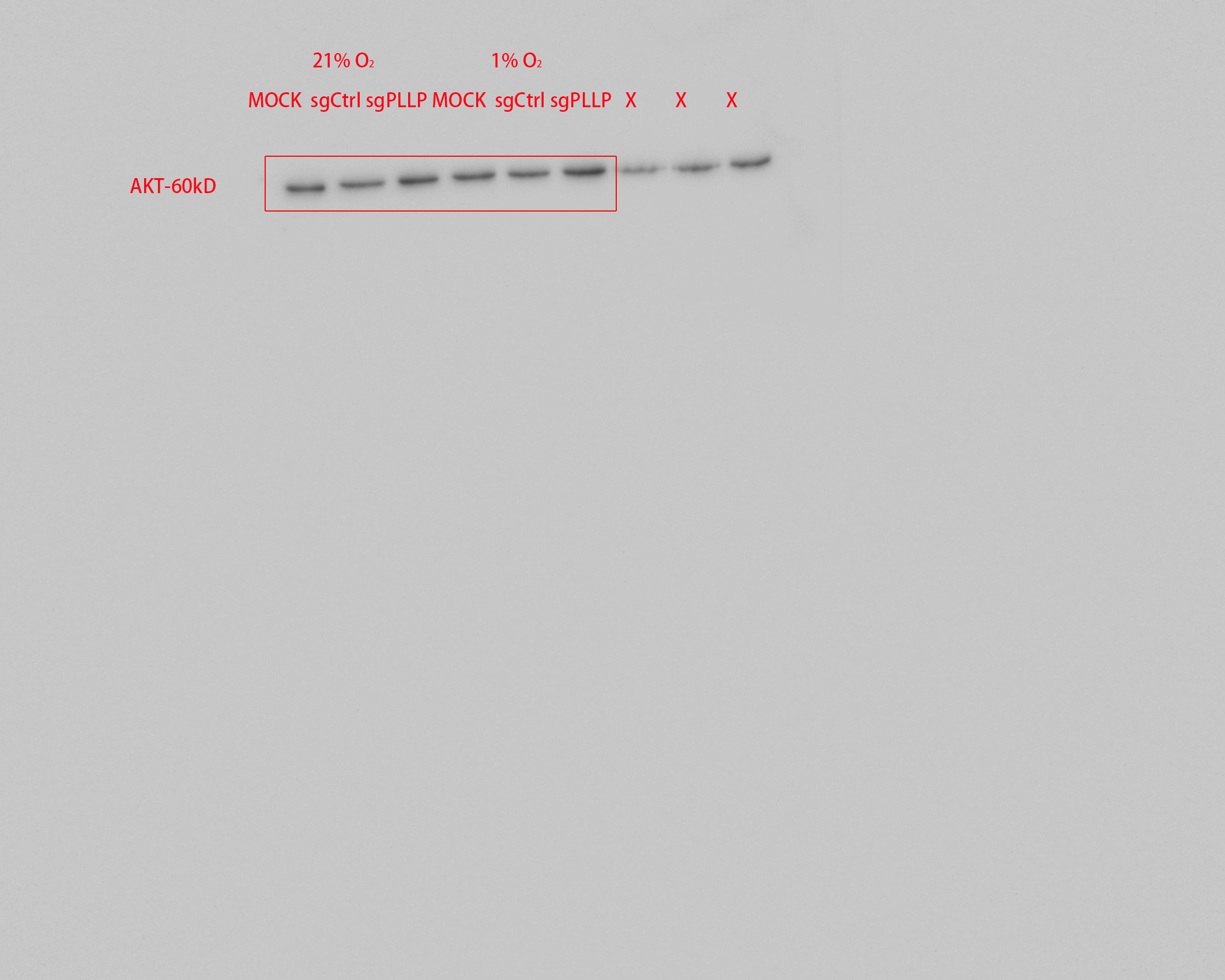

Supplement: Supplementary file 1 — Original Images-WB [file 41420_2025_2526_MOESM1_ESM.zip › Original Images-WB/Fig6F-AKT.tif]

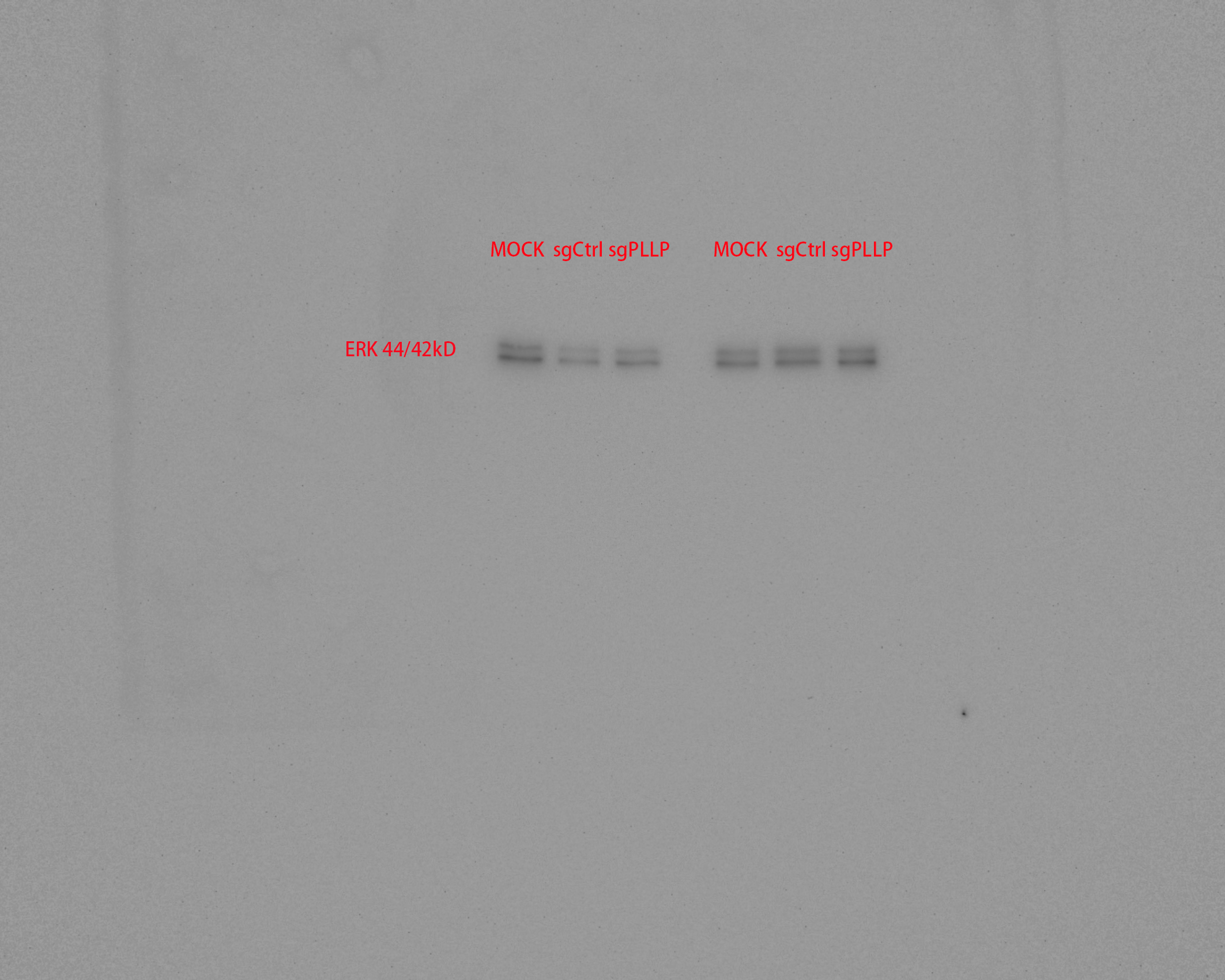

Supplement: Supplementary file 1 — Original Images-WB [file 41420_2025_2526_MOESM1_ESM.zip › Original Images-WB/Fig6F-ERK-bev.tif]

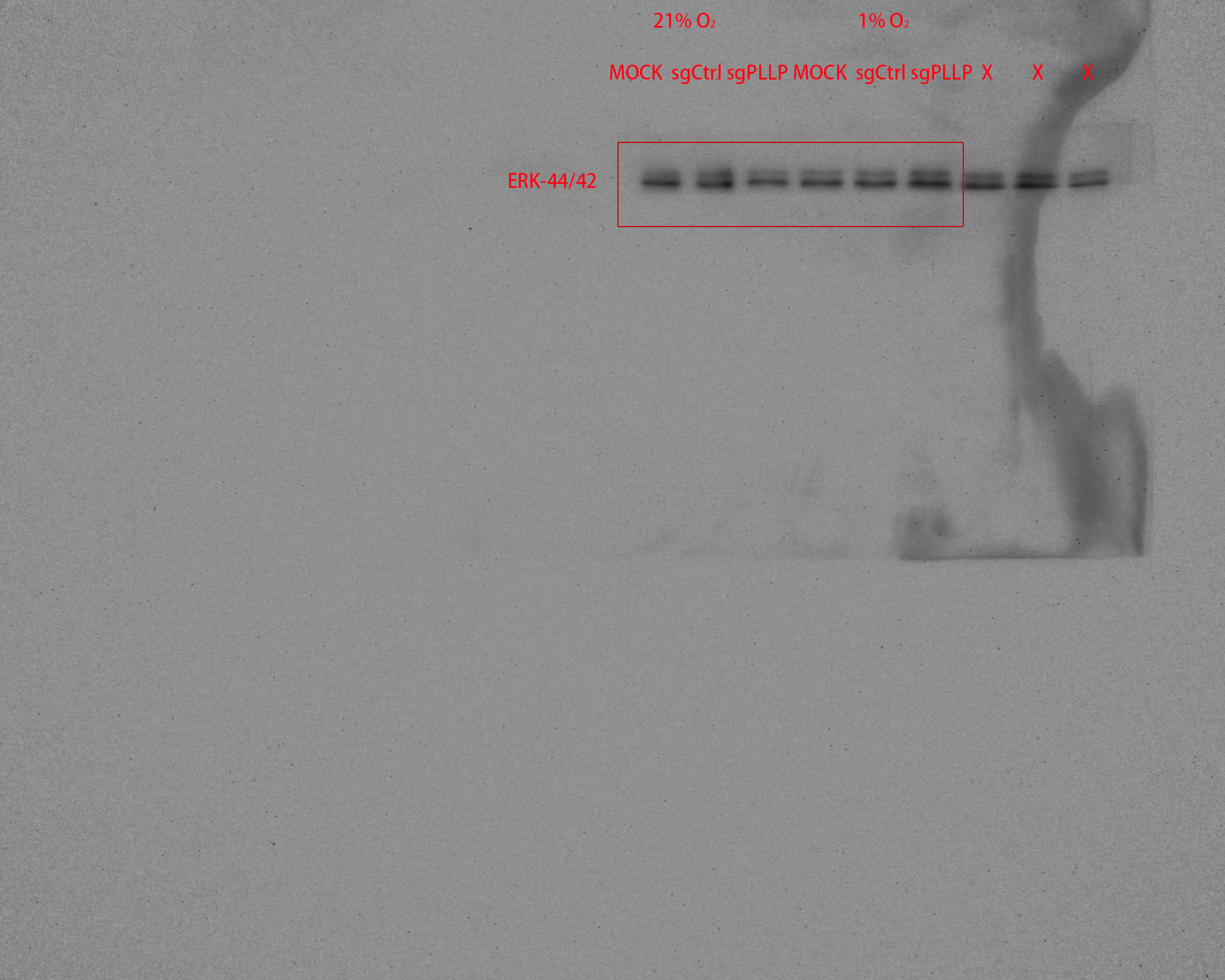

Supplement: Supplementary file 1 — Original Images-WB [file 41420_2025_2526_MOESM1_ESM.zip › Original Images-WB/Fig6F-ERK.tif]

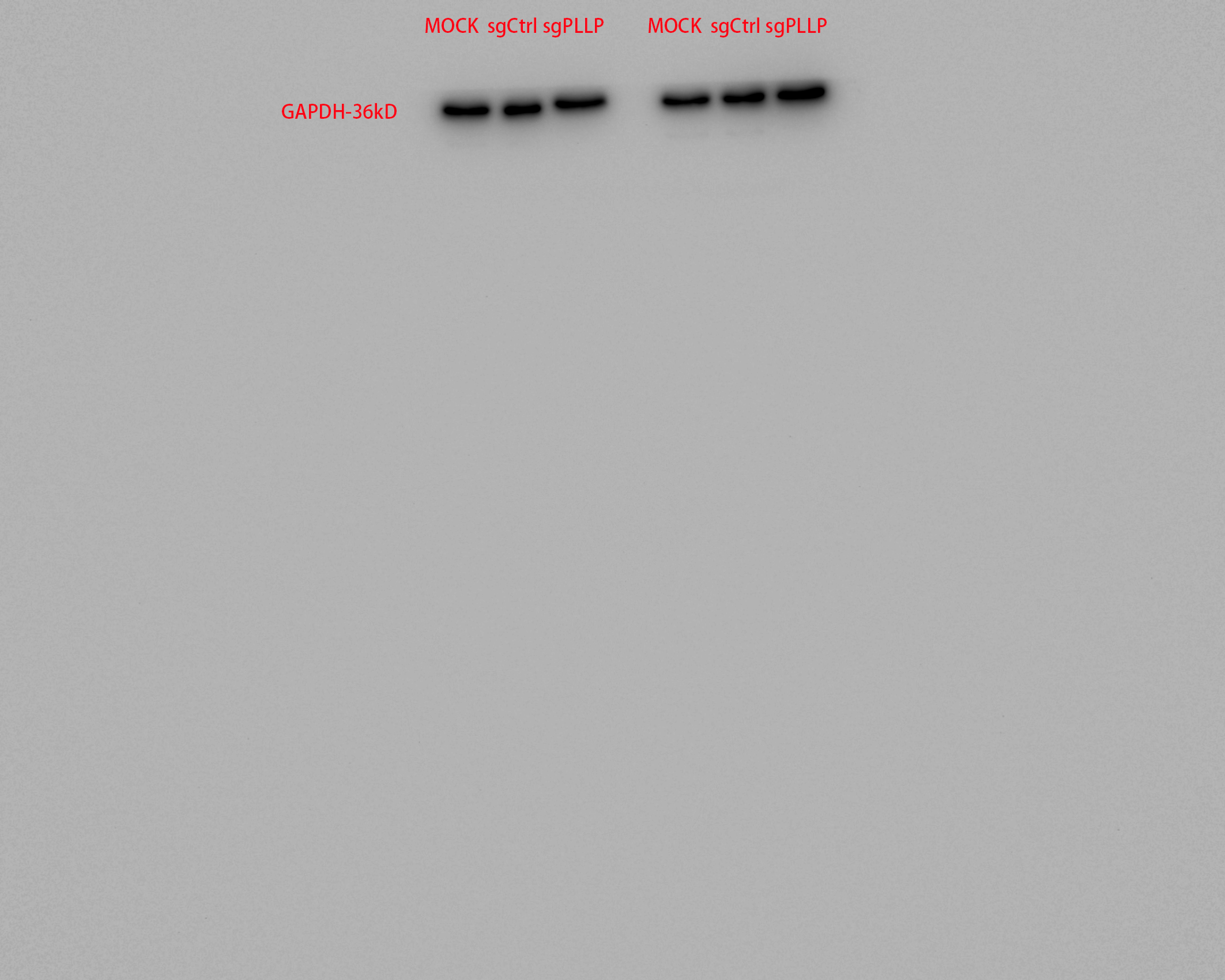

Supplement: Supplementary file 1 — Original Images-WB [file 41420_2025_2526_MOESM1_ESM.zip › Original Images-WB/Fig6F-GAPDH-bev.tif]

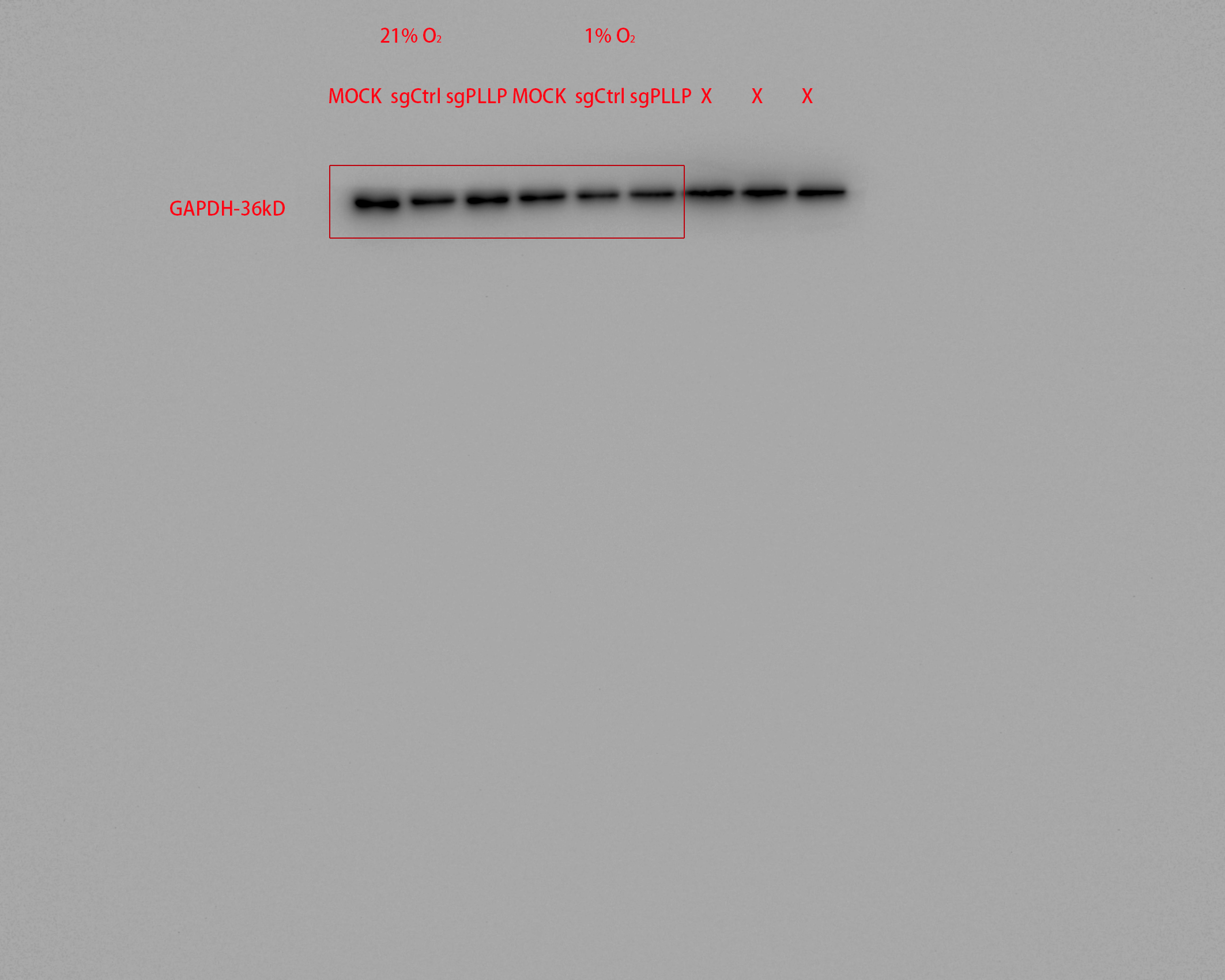

Supplement: Supplementary file 1 — Original Images-WB [file 41420_2025_2526_MOESM1_ESM.zip › Original Images-WB/Fig6F-GAPDH.tif]

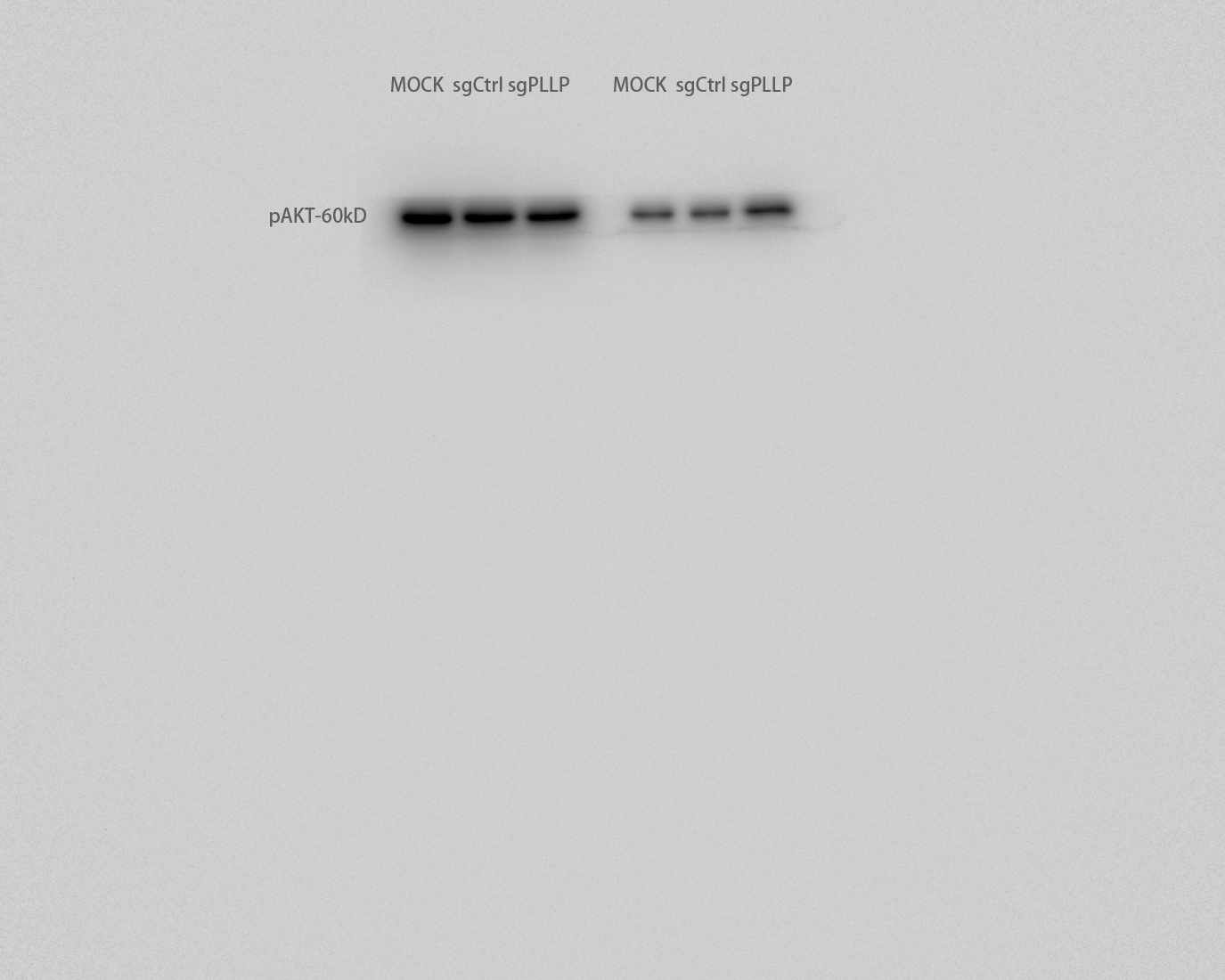

Supplement: Supplementary file 1 — Original Images-WB [file 41420_2025_2526_MOESM1_ESM.zip › Original Images-WB/Fig6F-pAKT-bev.tif]

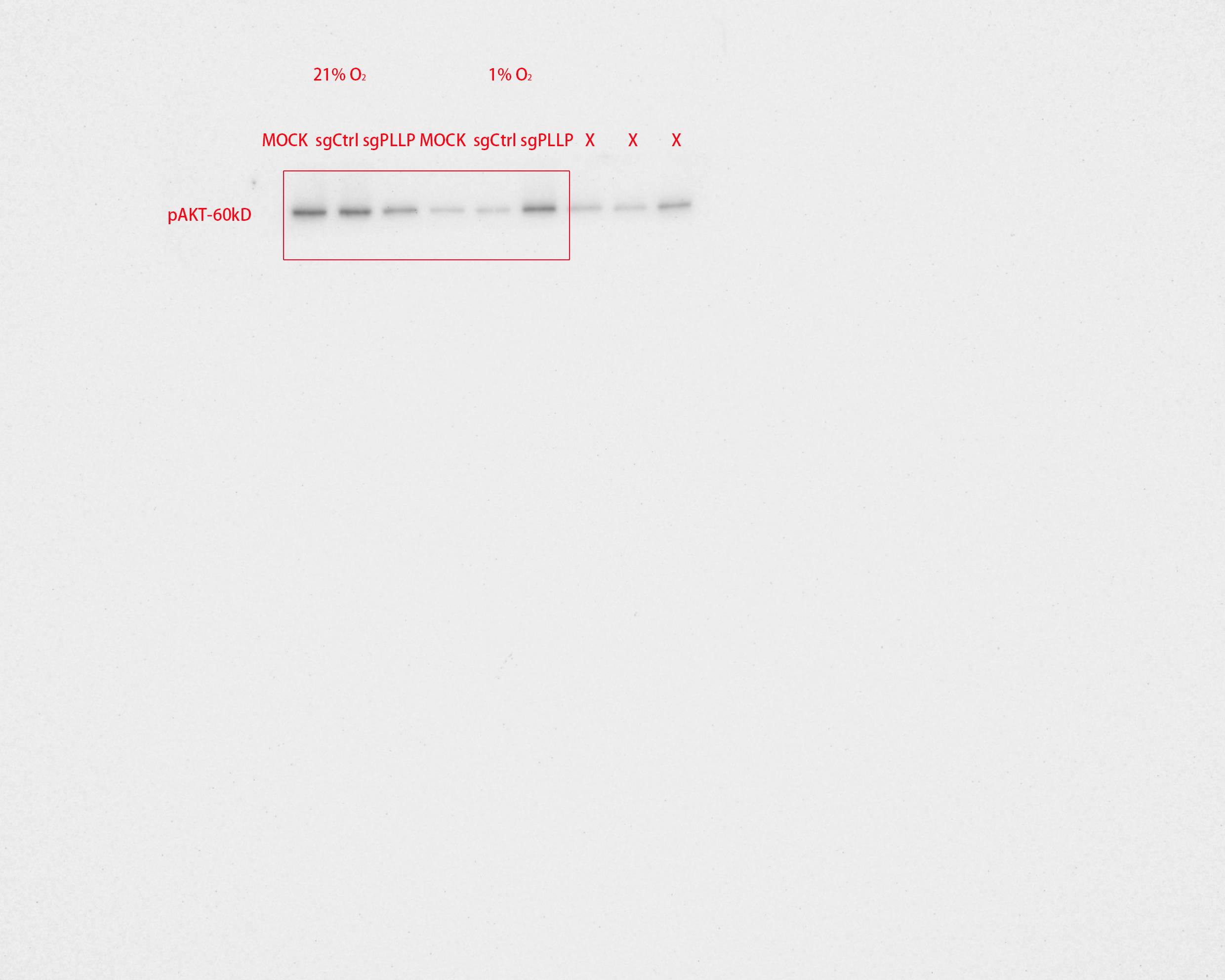

Supplement: Supplementary file 1 — Original Images-WB [file 41420_2025_2526_MOESM1_ESM.zip › Original Images-WB/Fig6F-pAKT.tif]

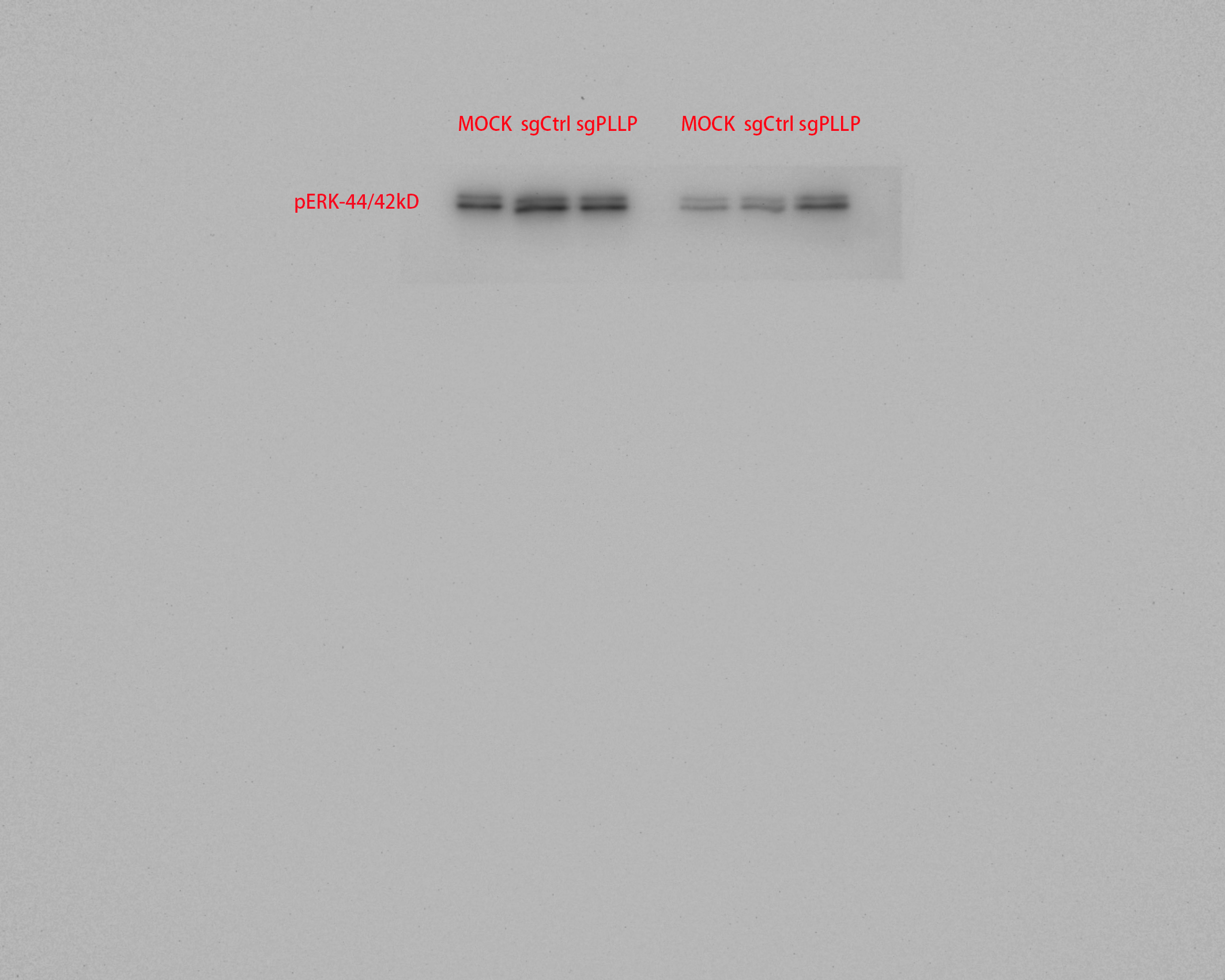

Supplement: Supplementary file 1 — Original Images-WB [file 41420_2025_2526_MOESM1_ESM.zip › Original Images-WB/Fig6F-pERK-bev.tif]

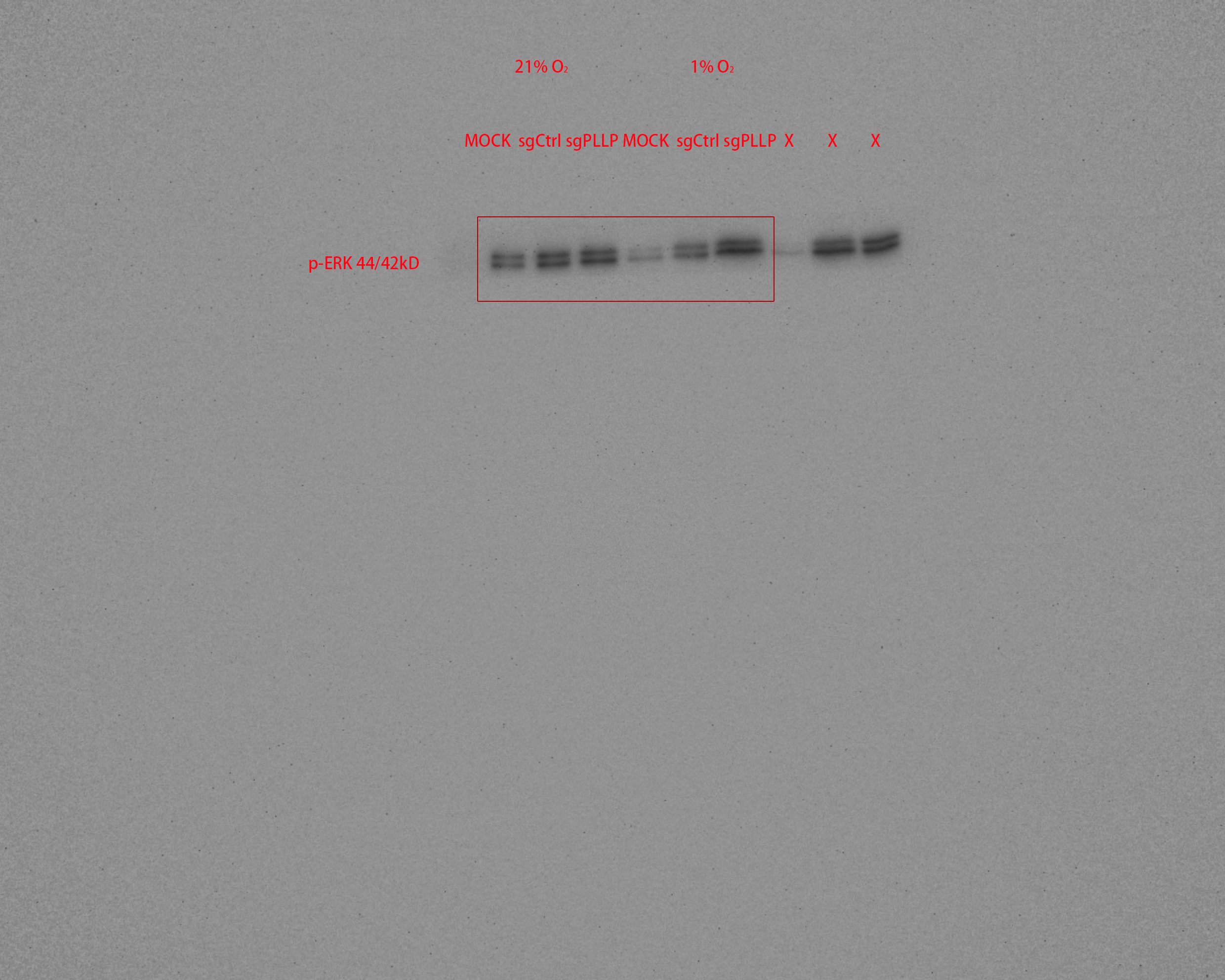

Supplement: Supplementary file 1 — Original Images-WB [file 41420_2025_2526_MOESM1_ESM.zip › Original Images-WB/Fig6F-pERK.tif]

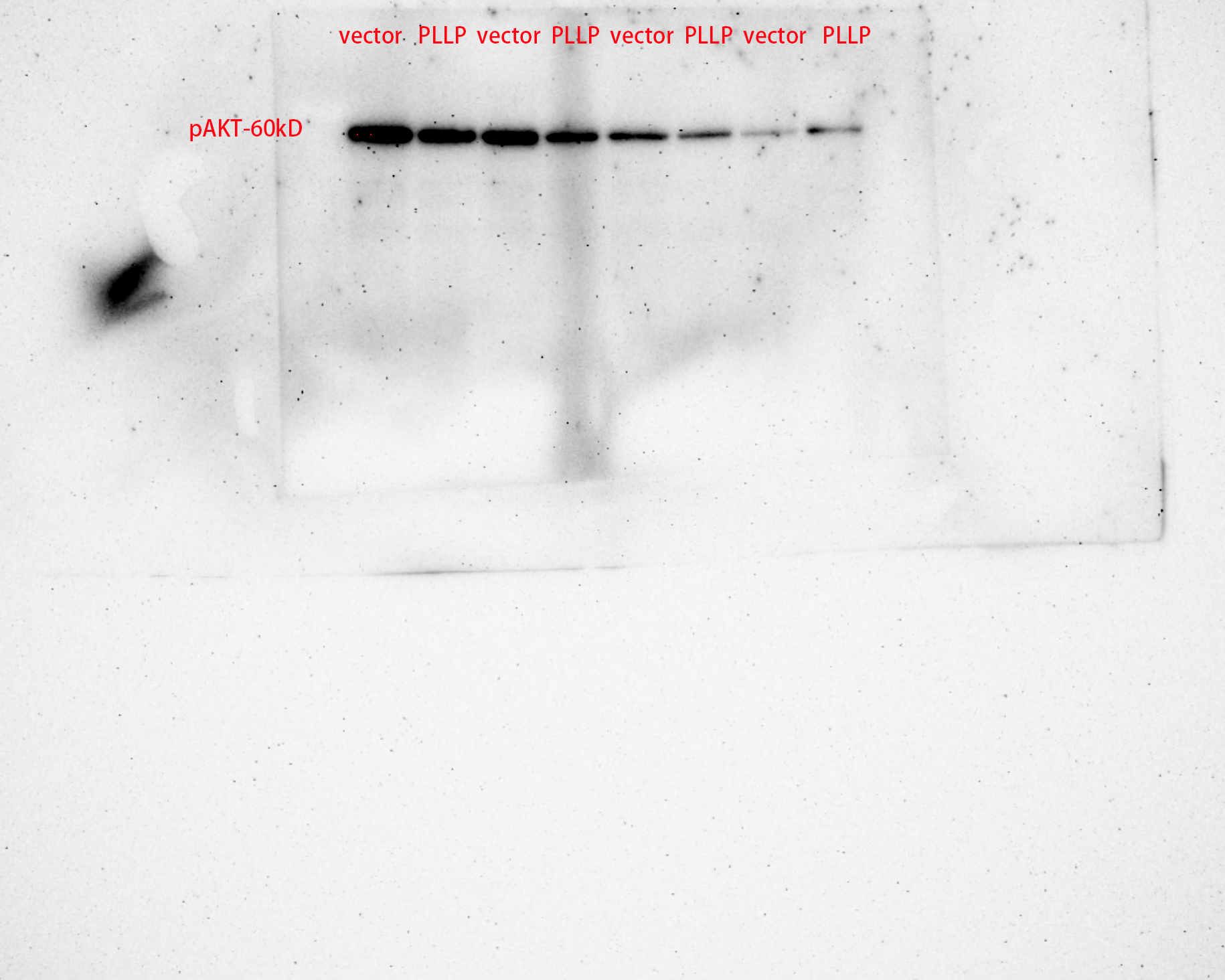

Supplement: Supplementary file 1 — Original Images-WB [file 41420_2025_2526_MOESM1_ESM.zip › Original Images-WB/Fig6G--pAKT.tif]

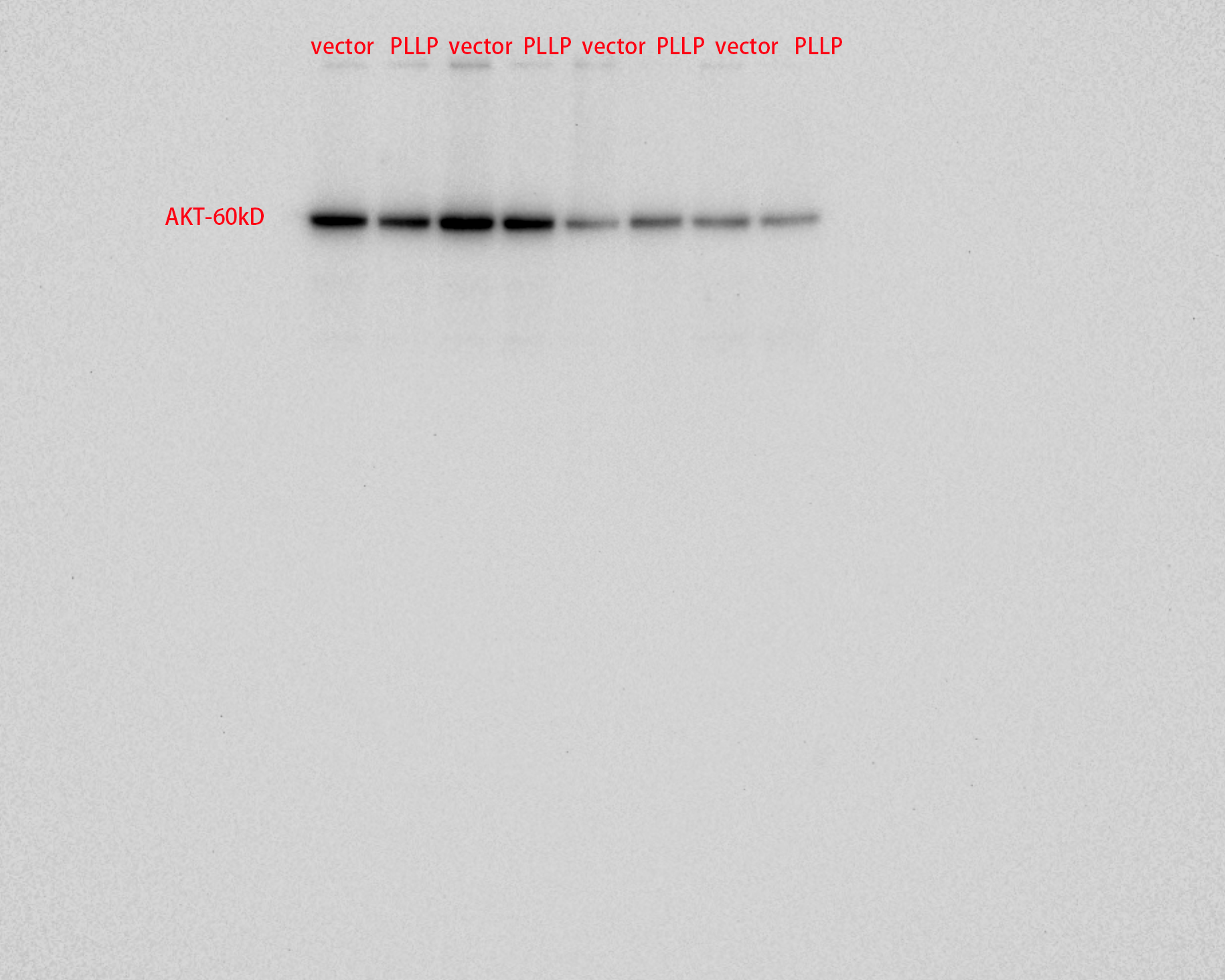

Supplement: Supplementary file 1 — Original Images-WB [file 41420_2025_2526_MOESM1_ESM.zip › Original Images-WB/Fig6G-AKT.tif]

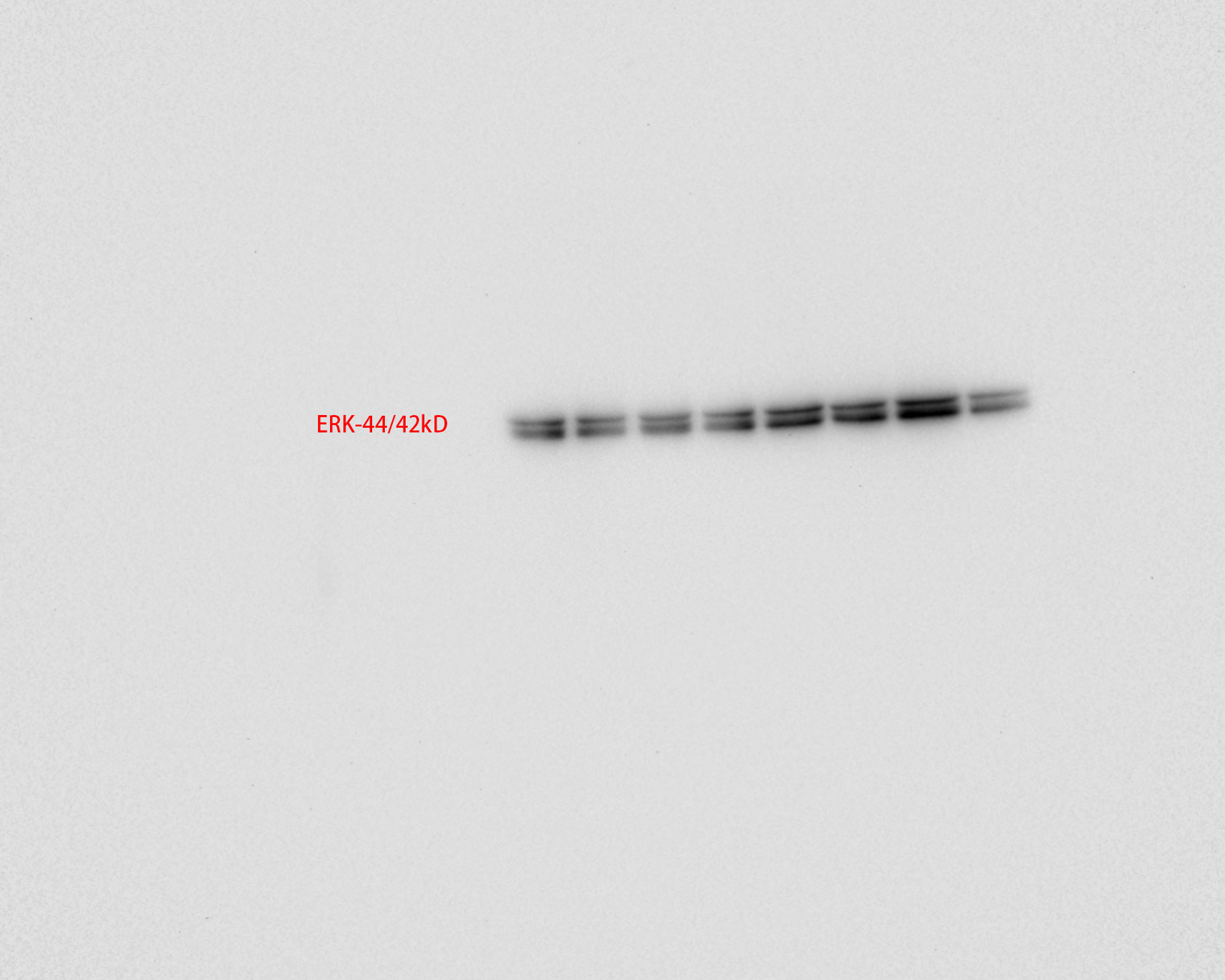

Supplement: Supplementary file 1 — Original Images-WB [file 41420_2025_2526_MOESM1_ESM.zip › Original Images-WB/Fig6G-ERK.tif]

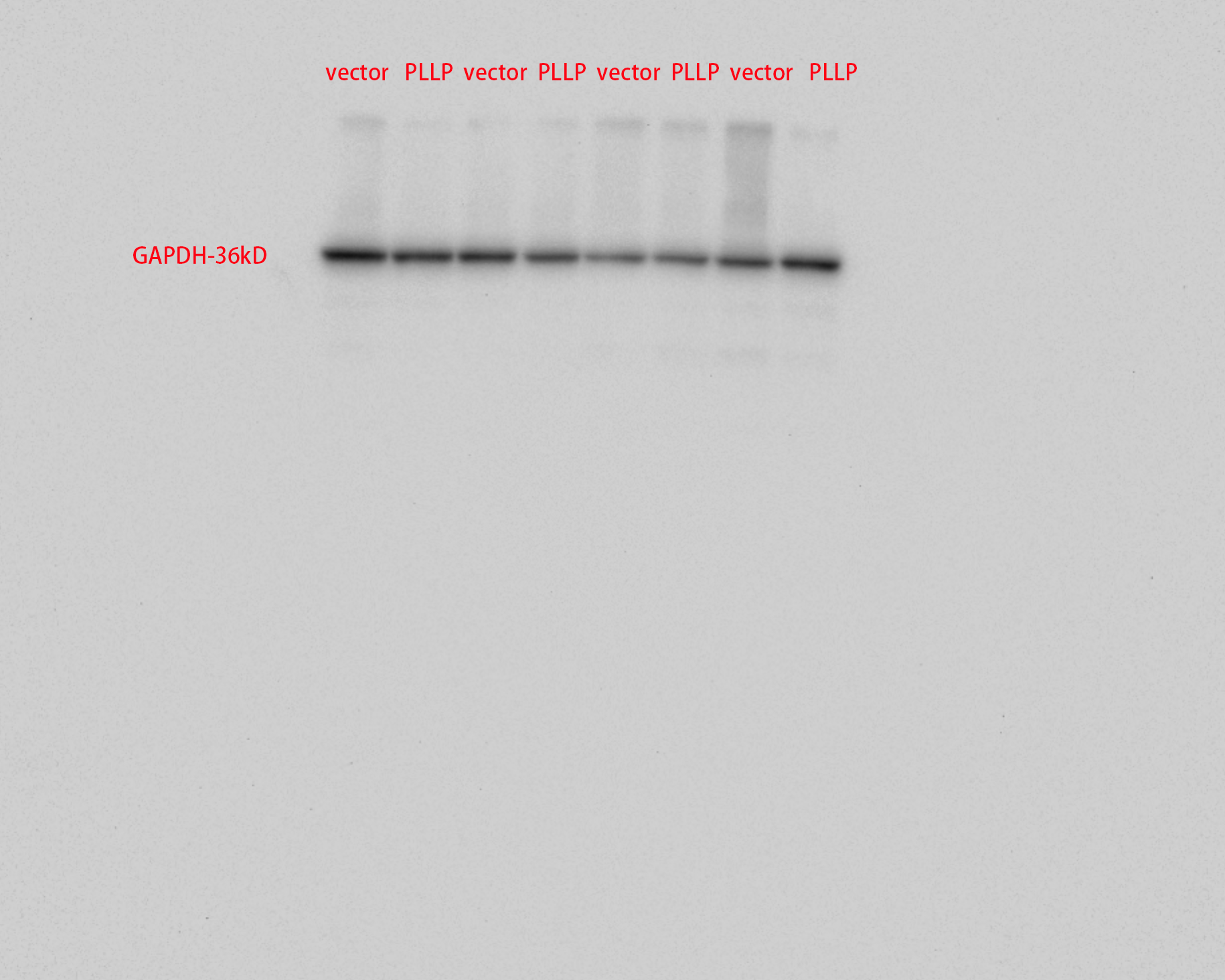

Supplement: Supplementary file 1 — Original Images-WB [file 41420_2025_2526_MOESM1_ESM.zip › Original Images-WB/Fig6G-GAPDH.tif]

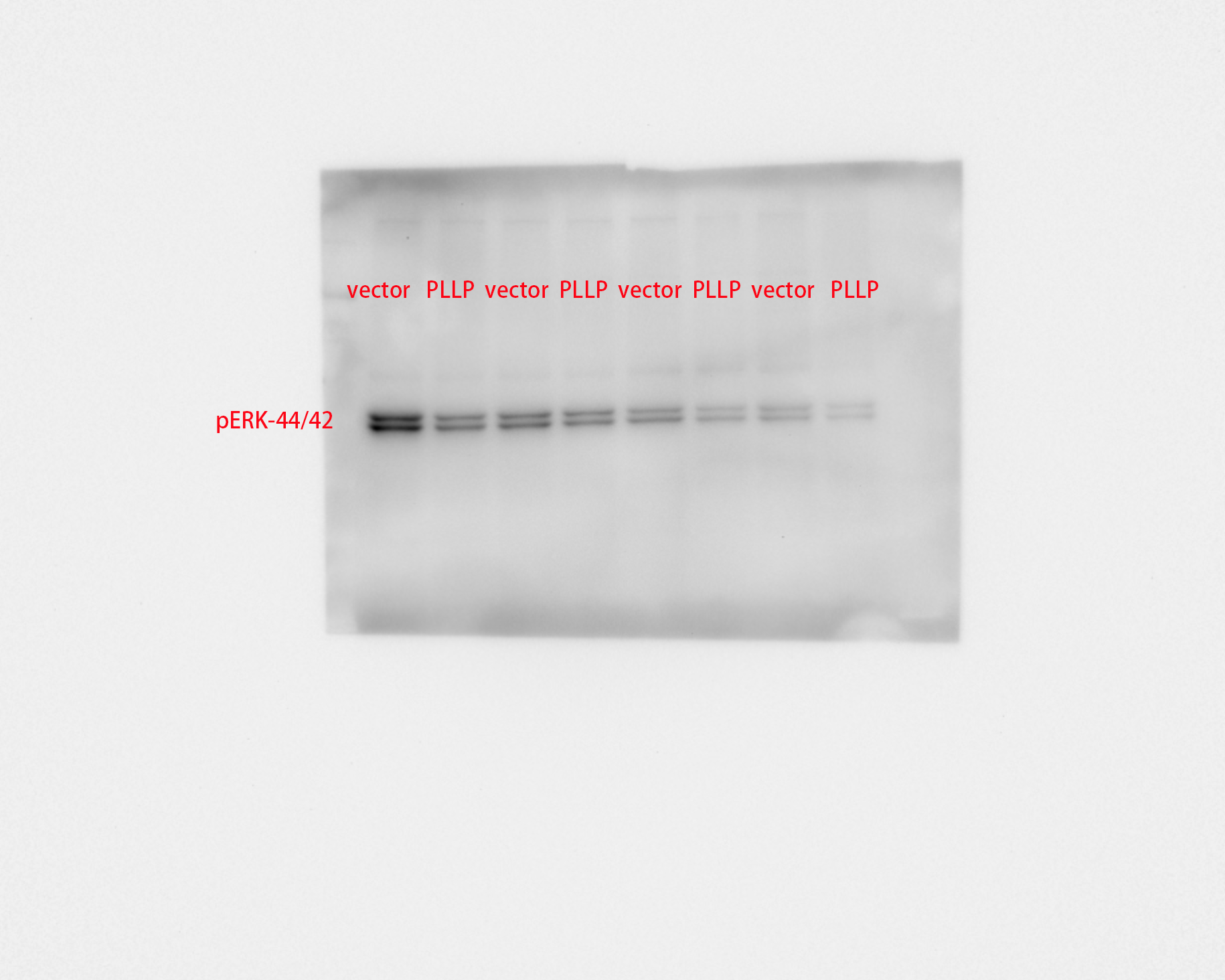

Supplement: Supplementary file 1 — Original Images-WB [file 41420_2025_2526_MOESM1_ESM.zip › Original Images-WB/Fig6G-pERK.tif]

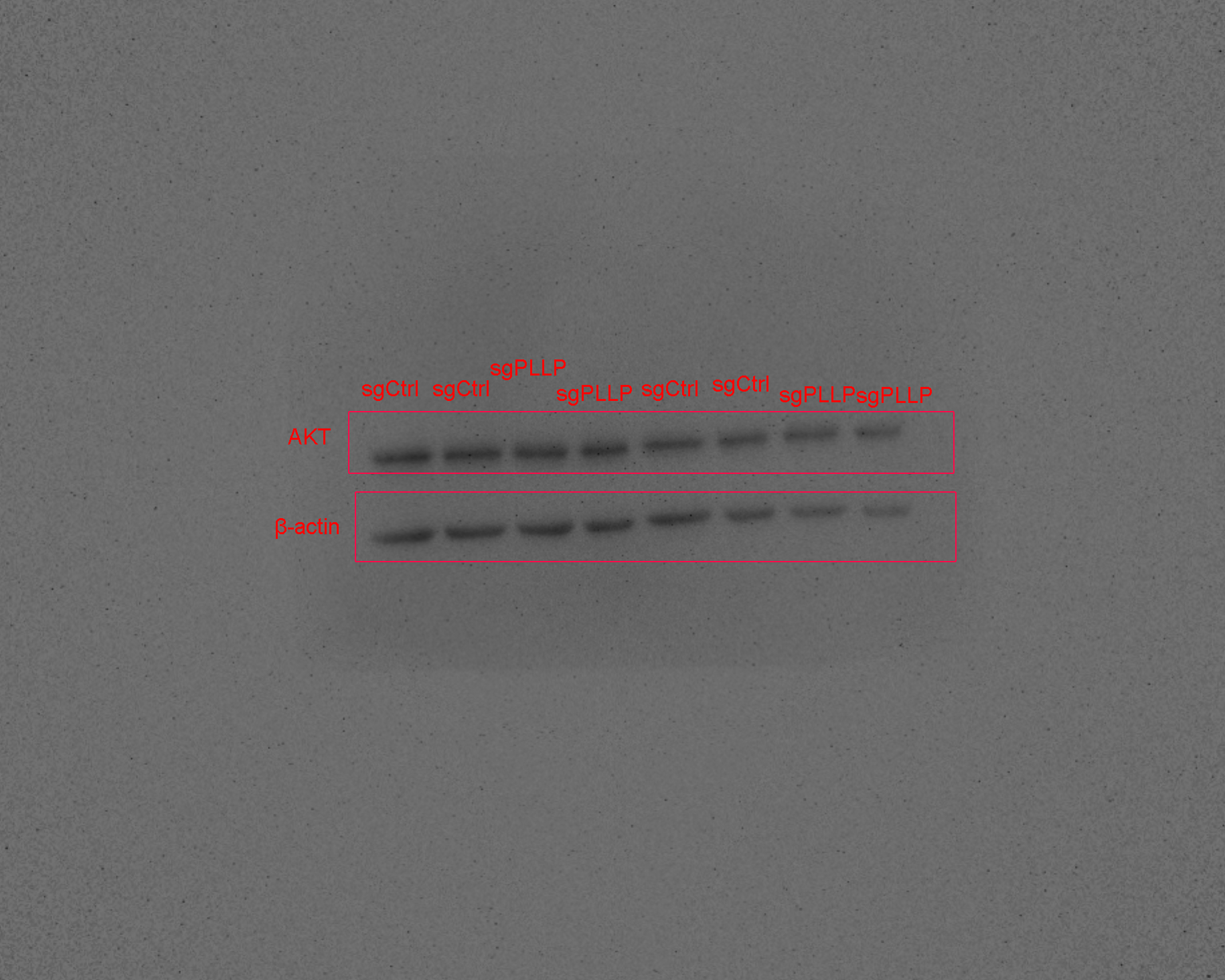

Supplement: Supplementary file 1 — Original Images-WB [file 41420_2025_2526_MOESM1_ESM.zip › Original Images-WB/Fig6H-AKT β-actin.tif]

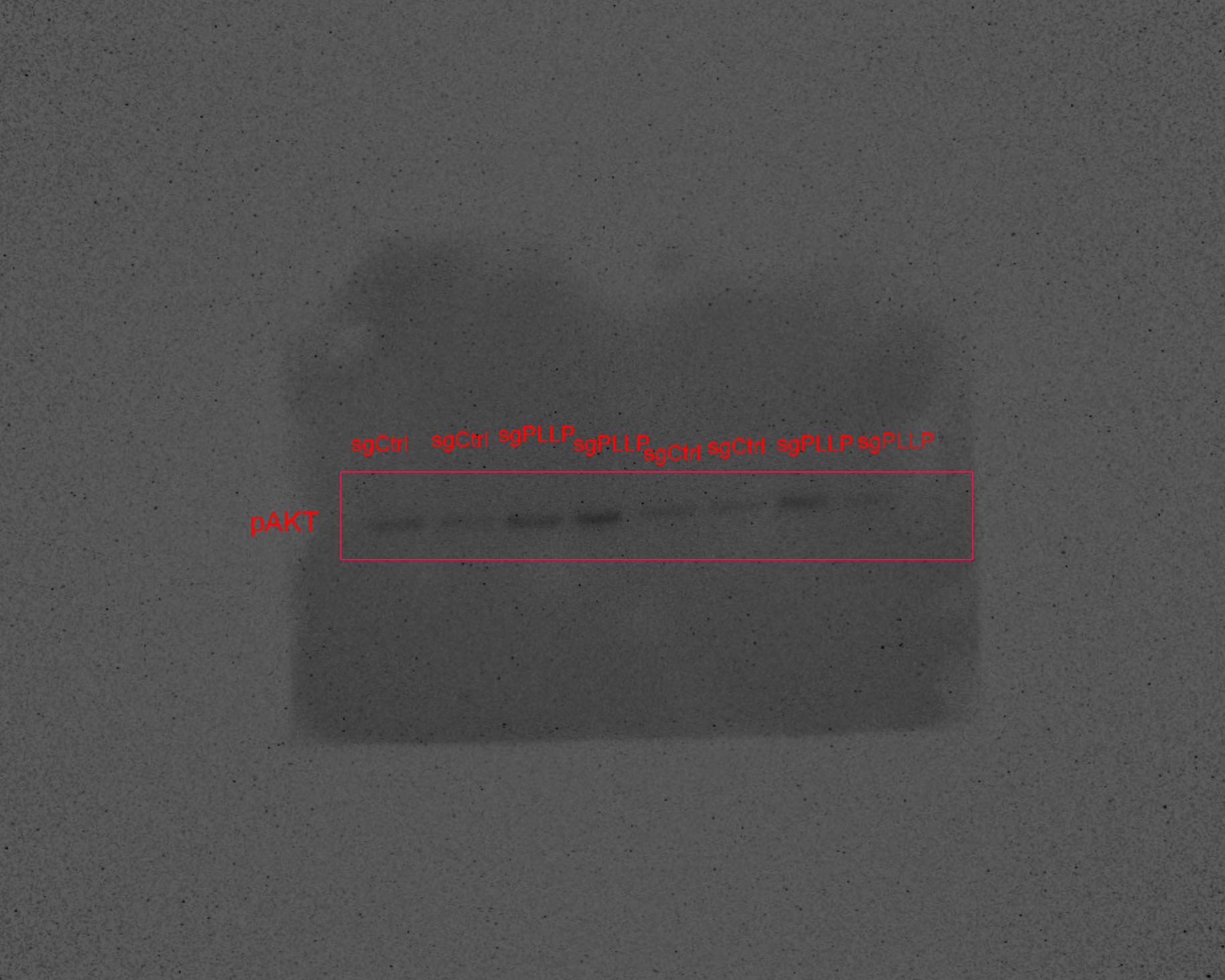

Supplement: Supplementary file 1 — Original Images-WB [file 41420_2025_2526_MOESM1_ESM.zip › Original Images-WB/Fig6H-pAKT.tif]

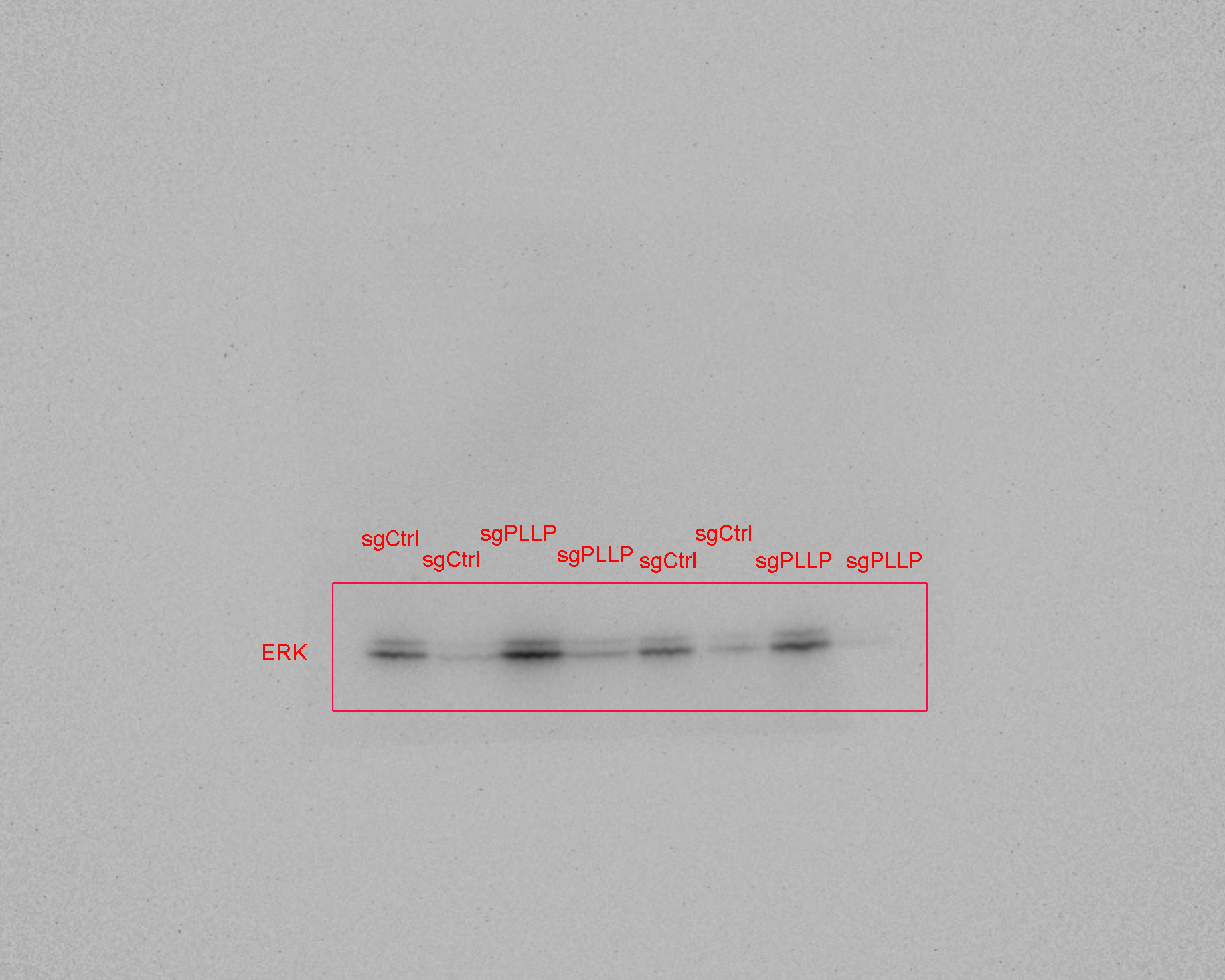

Supplement: Supplementary file 1 — Original Images-WB [file 41420_2025_2526_MOESM1_ESM.zip › Original Images-WB/Fig6I-ERK.tif]

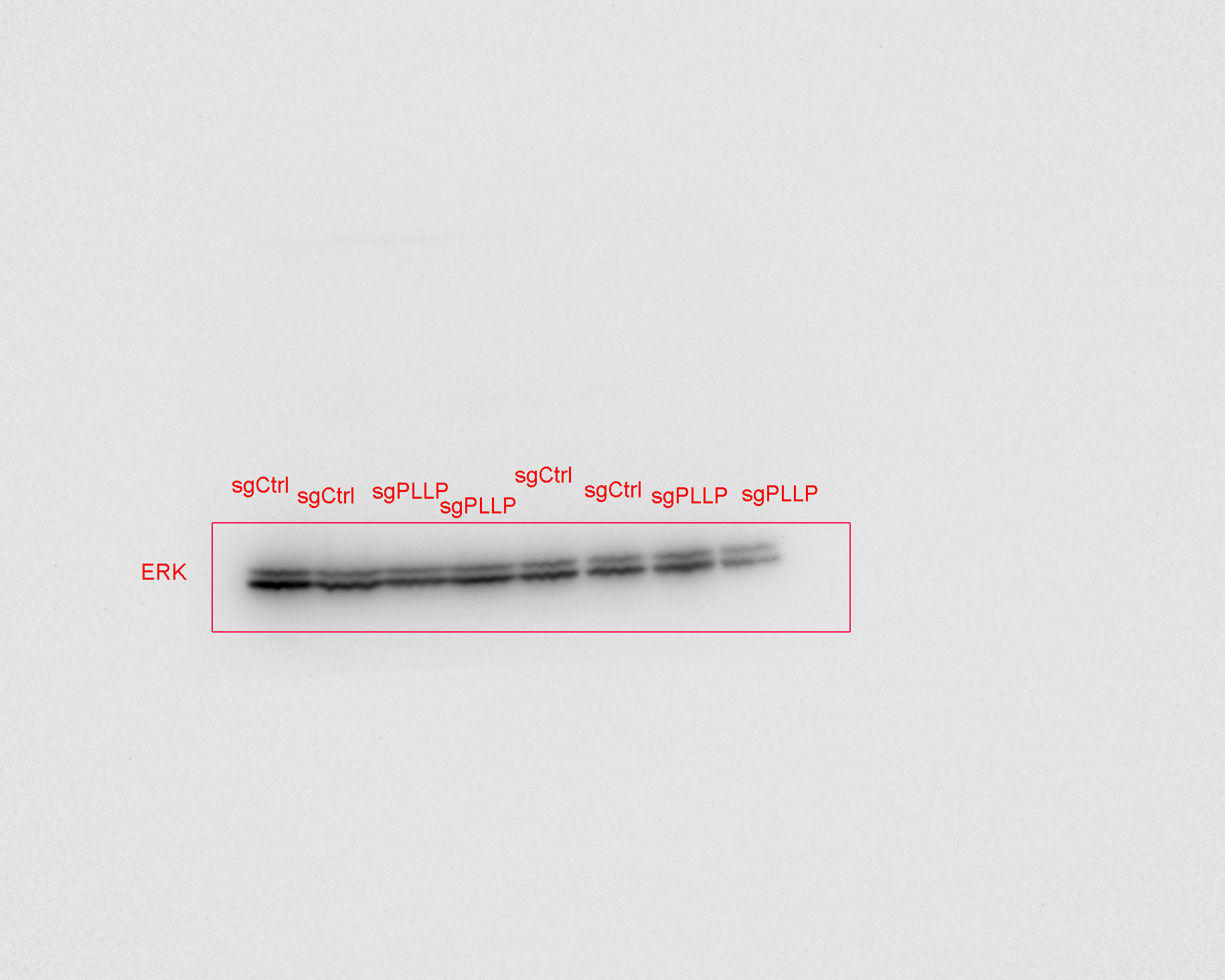

Supplement: Supplementary file 1 — Original Images-WB [file 41420_2025_2526_MOESM1_ESM.zip › Original Images-WB/Fig6I-pERK.tif]

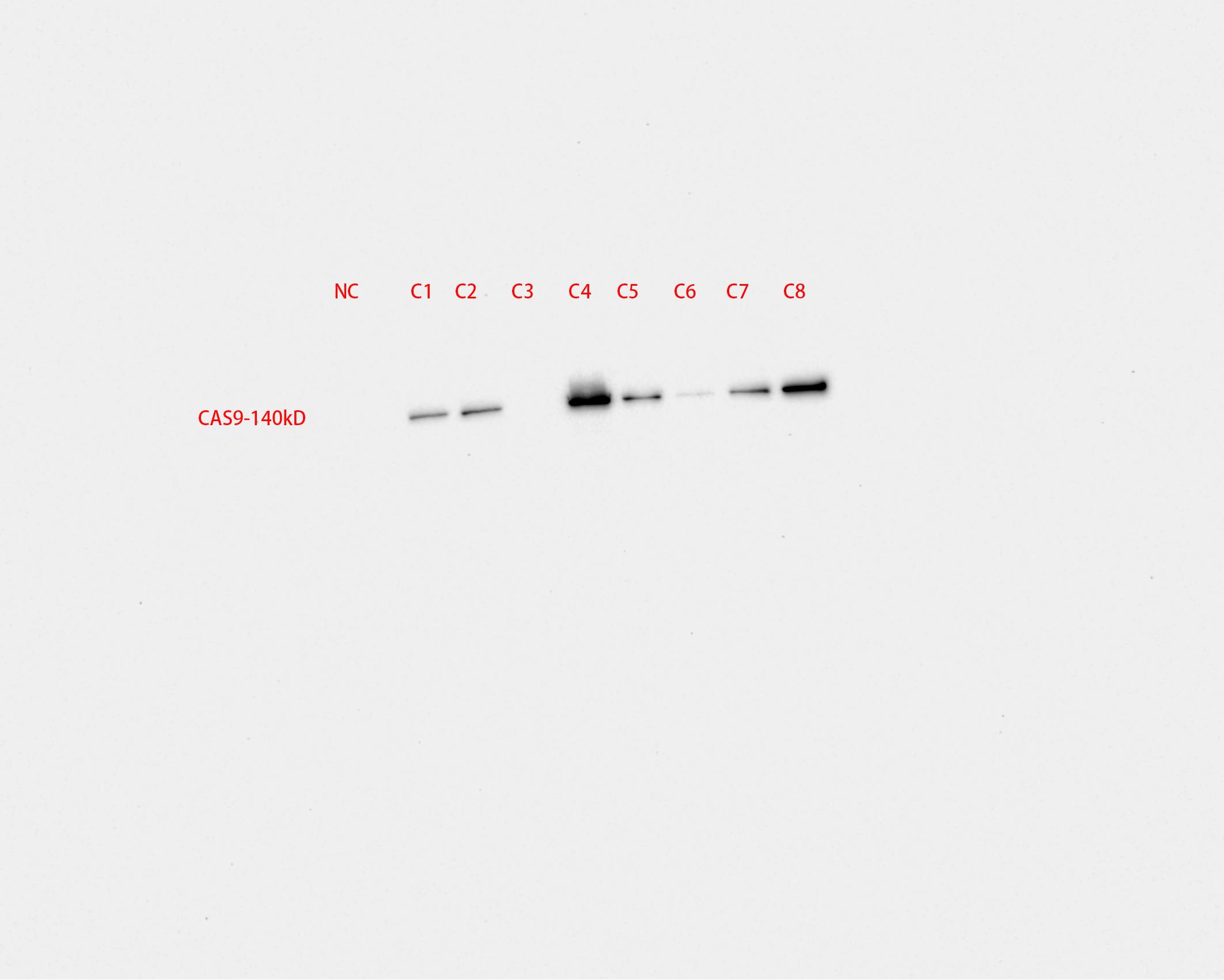

Supplement: Supplementary file 1 — Original Images-WB [file 41420_2025_2526_MOESM1_ESM.zip › Original Images-WB/FigS1D-CAS9.tif]

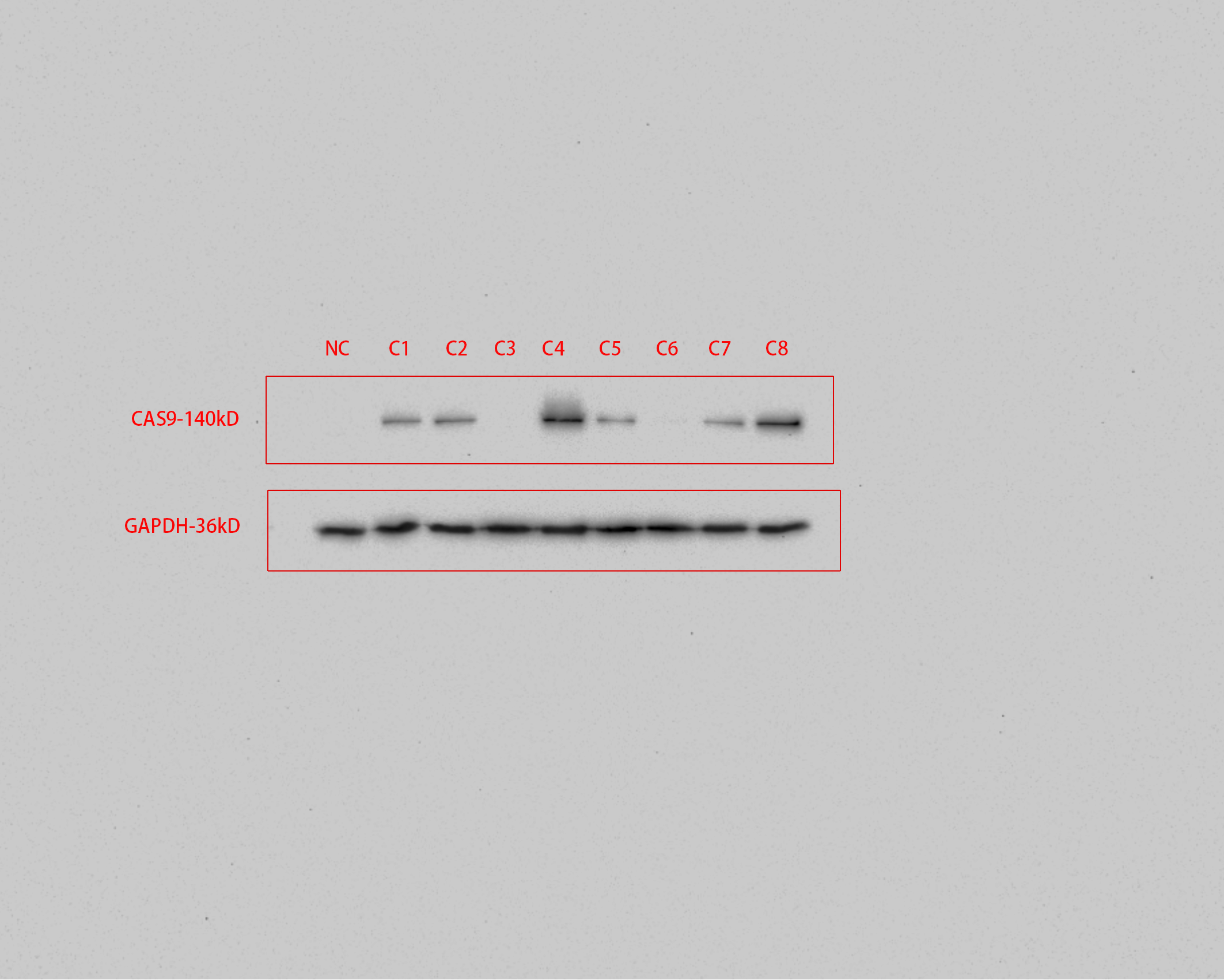

Supplement: Supplementary file 1 — Original Images-WB [file 41420_2025_2526_MOESM1_ESM.zip › Original Images-WB/FigS1D-GAPDH.tif]

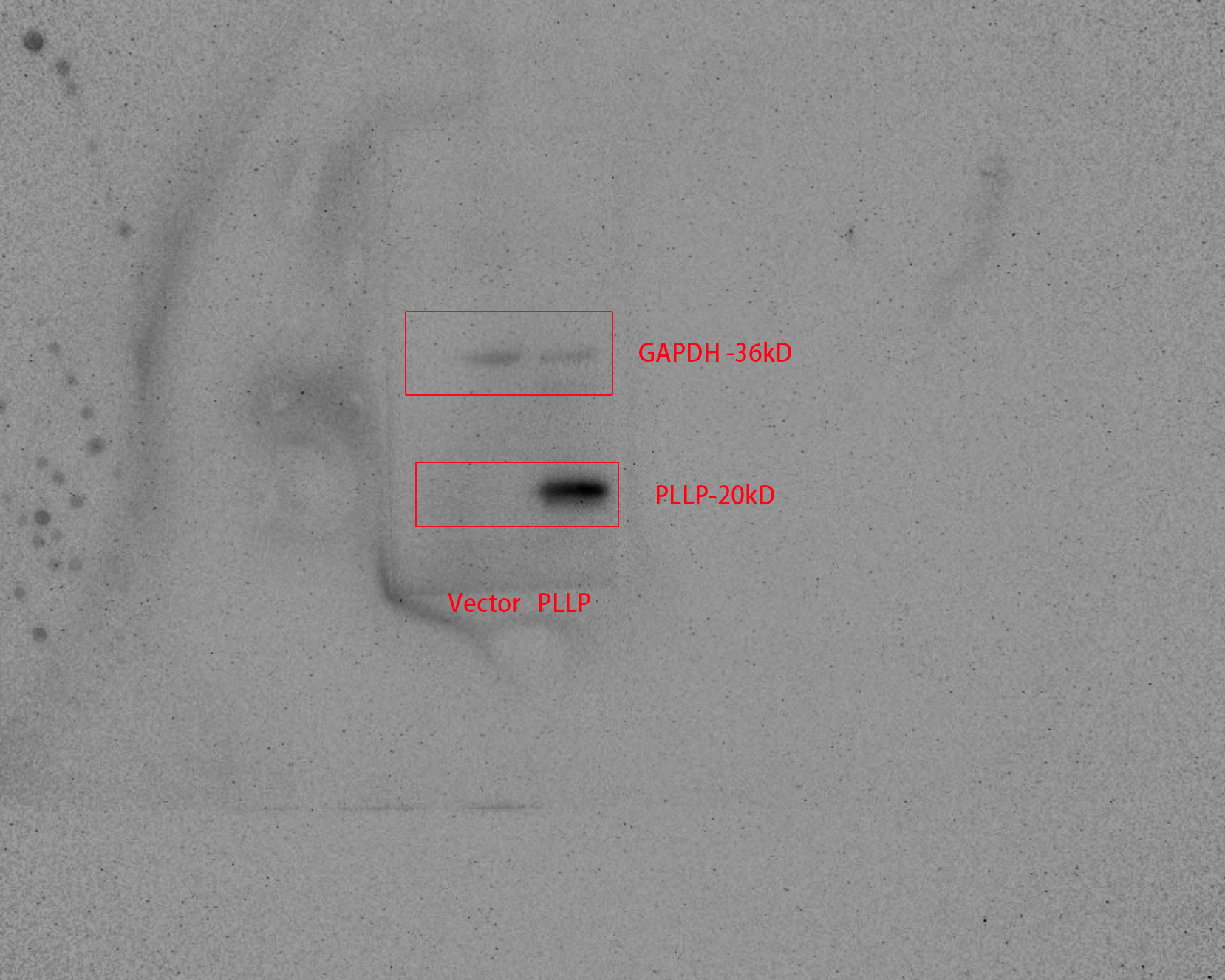

Supplement: Supplementary file 1 — Original Images-WB [file 41420_2025_2526_MOESM1_ESM.zip › Original Images-WB/FigS1J-GAPDH.tif]

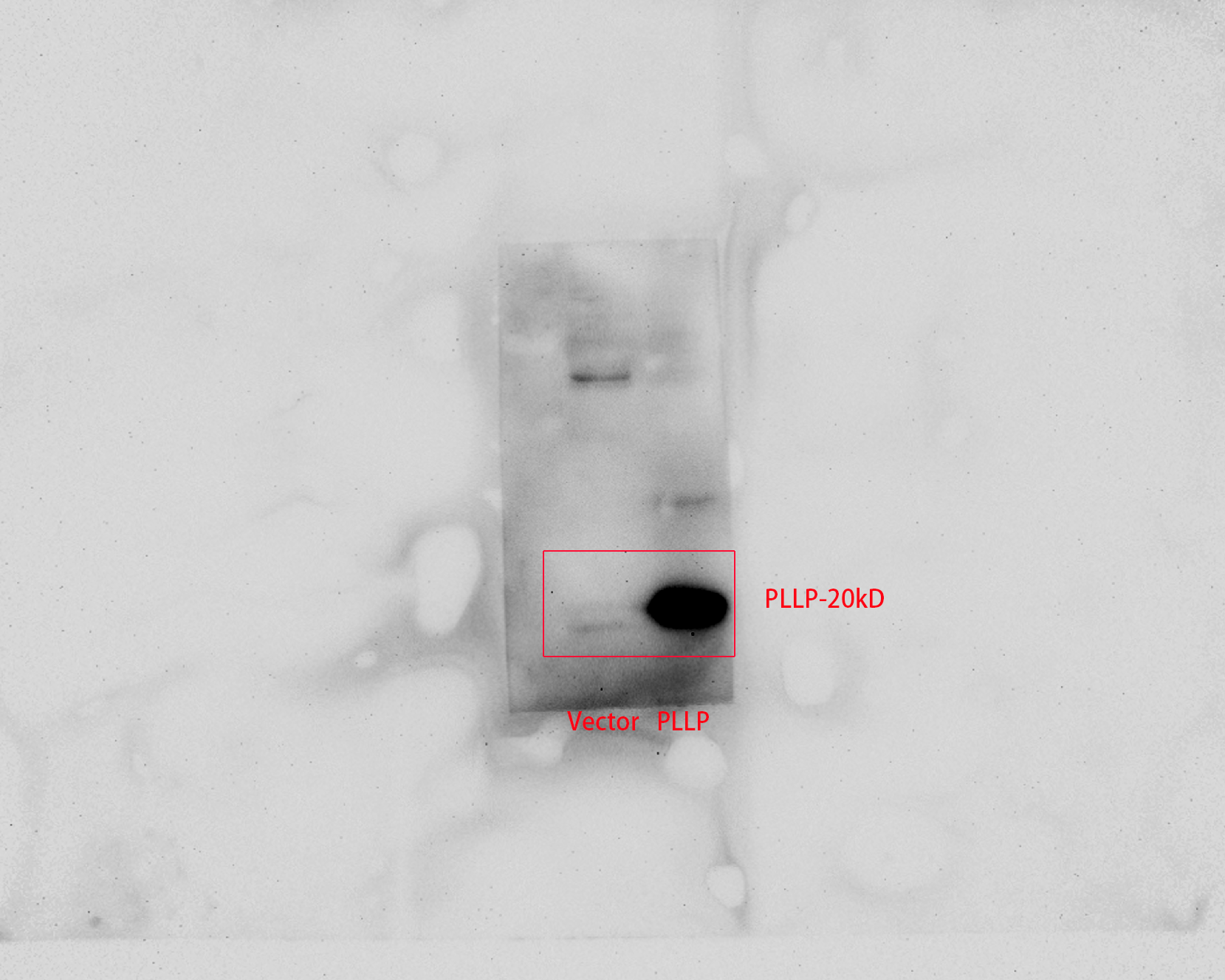

Supplement: Supplementary file 1 — Original Images-WB [file 41420_2025_2526_MOESM1_ESM.zip › Original Images-WB/FigS1J-PLLP.tif]

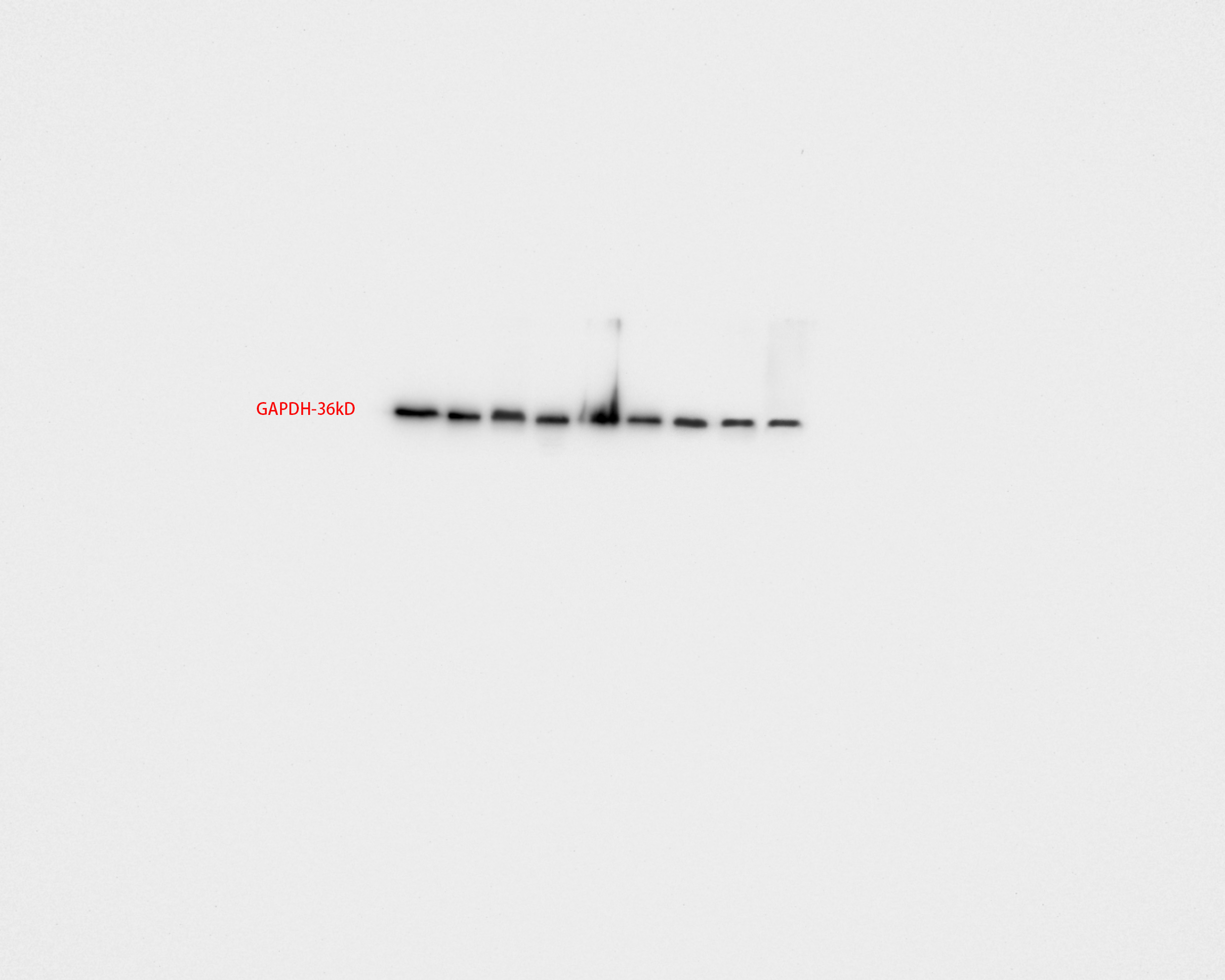

Supplement: Supplementary file 1 — Original Images-WB [file 41420_2025_2526_MOESM1_ESM.zip › Original Images-WB/FigS2A-GAPDH.tif]

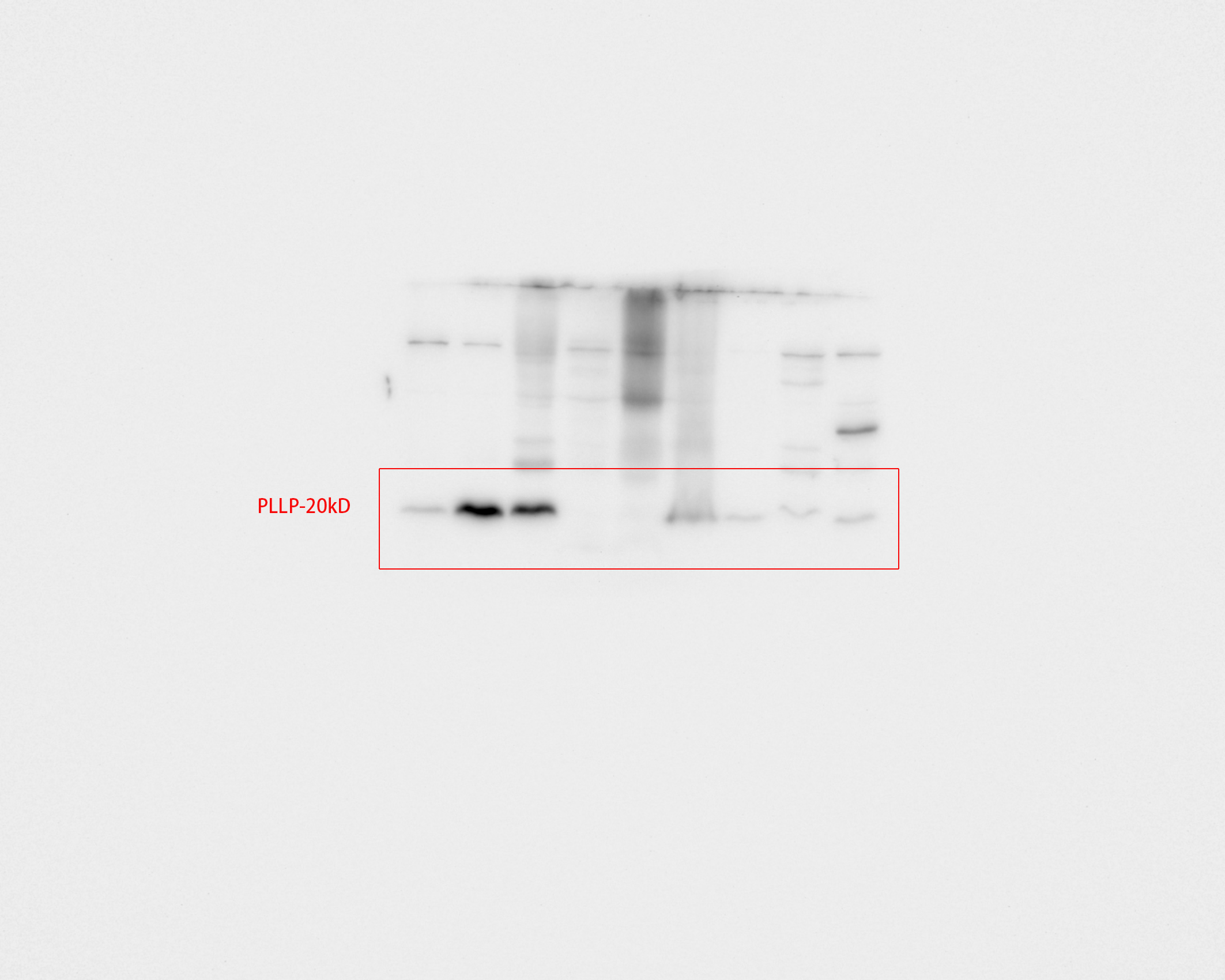

Supplement: Supplementary file 1 — Original Images-WB [file 41420_2025_2526_MOESM1_ESM.zip › Original Images-WB/FigS2A-PLLP.tif]

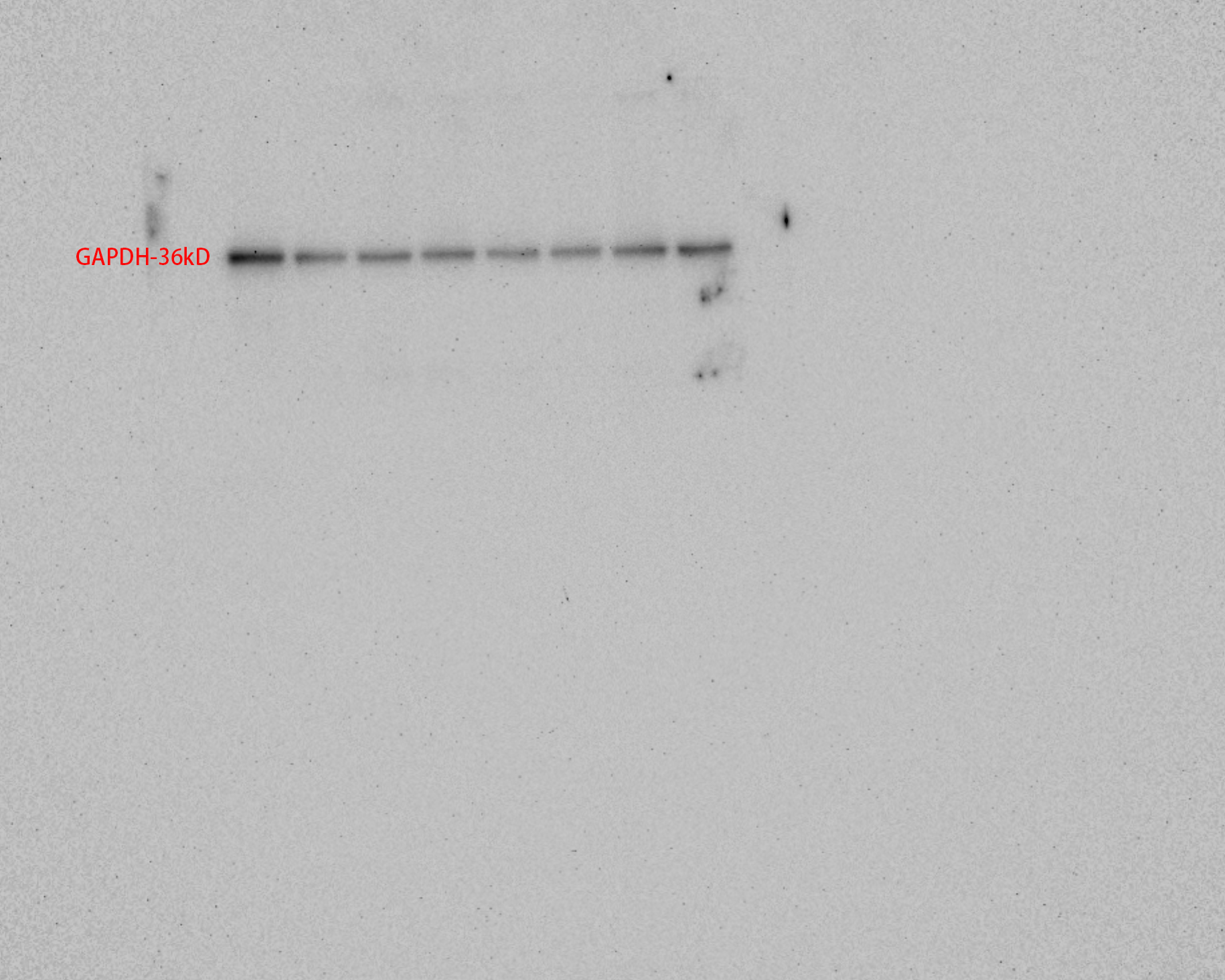

Supplement: Supplementary file 1 — Original Images-WB [file 41420_2025_2526_MOESM1_ESM.zip › Original Images-WB/FigS2B-GAPDH.tif]

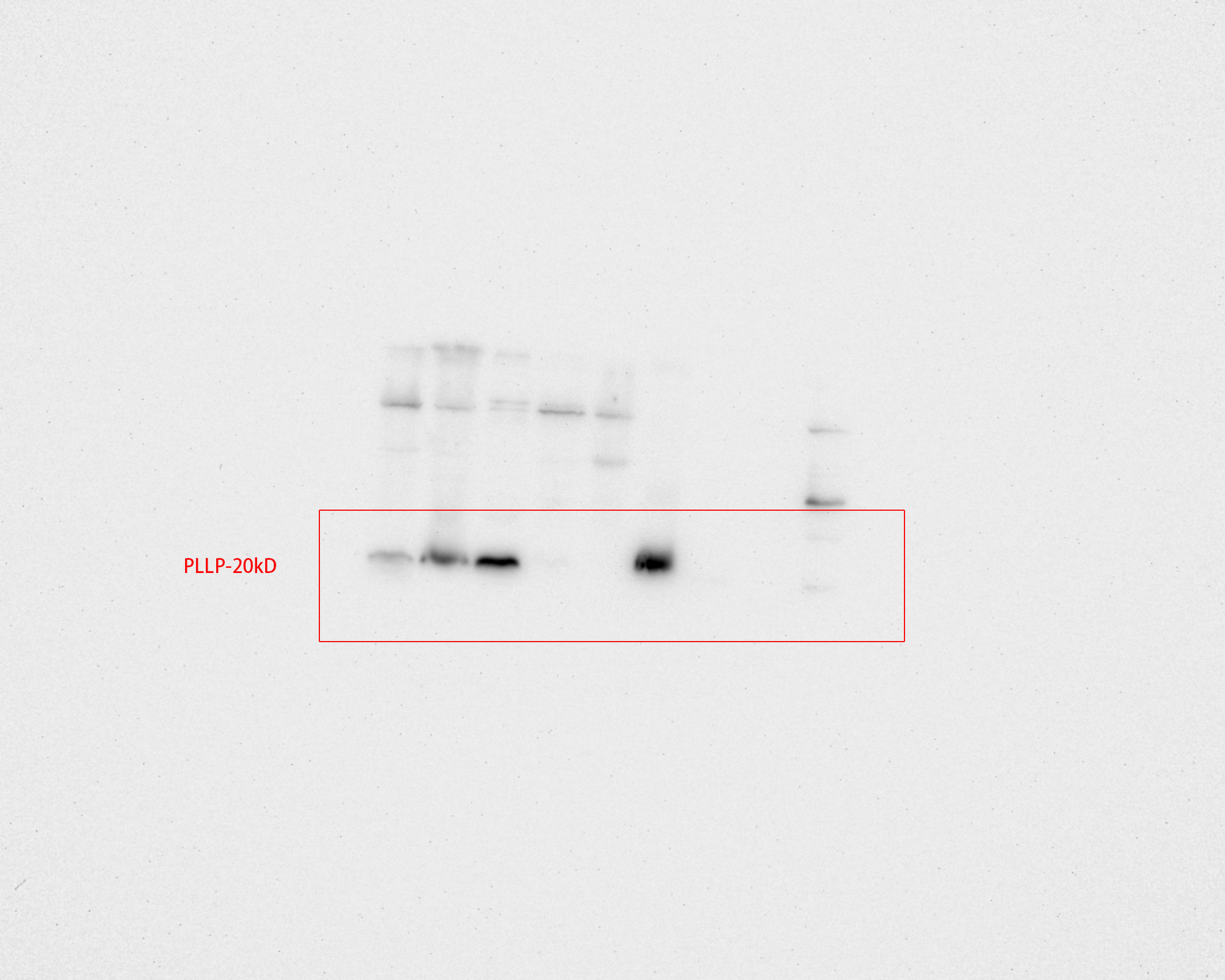

Supplement: Supplementary file 1 — Original Images-WB [file 41420_2025_2526_MOESM1_ESM.zip › Original Images-WB/FigS2B-PLLP.tif]

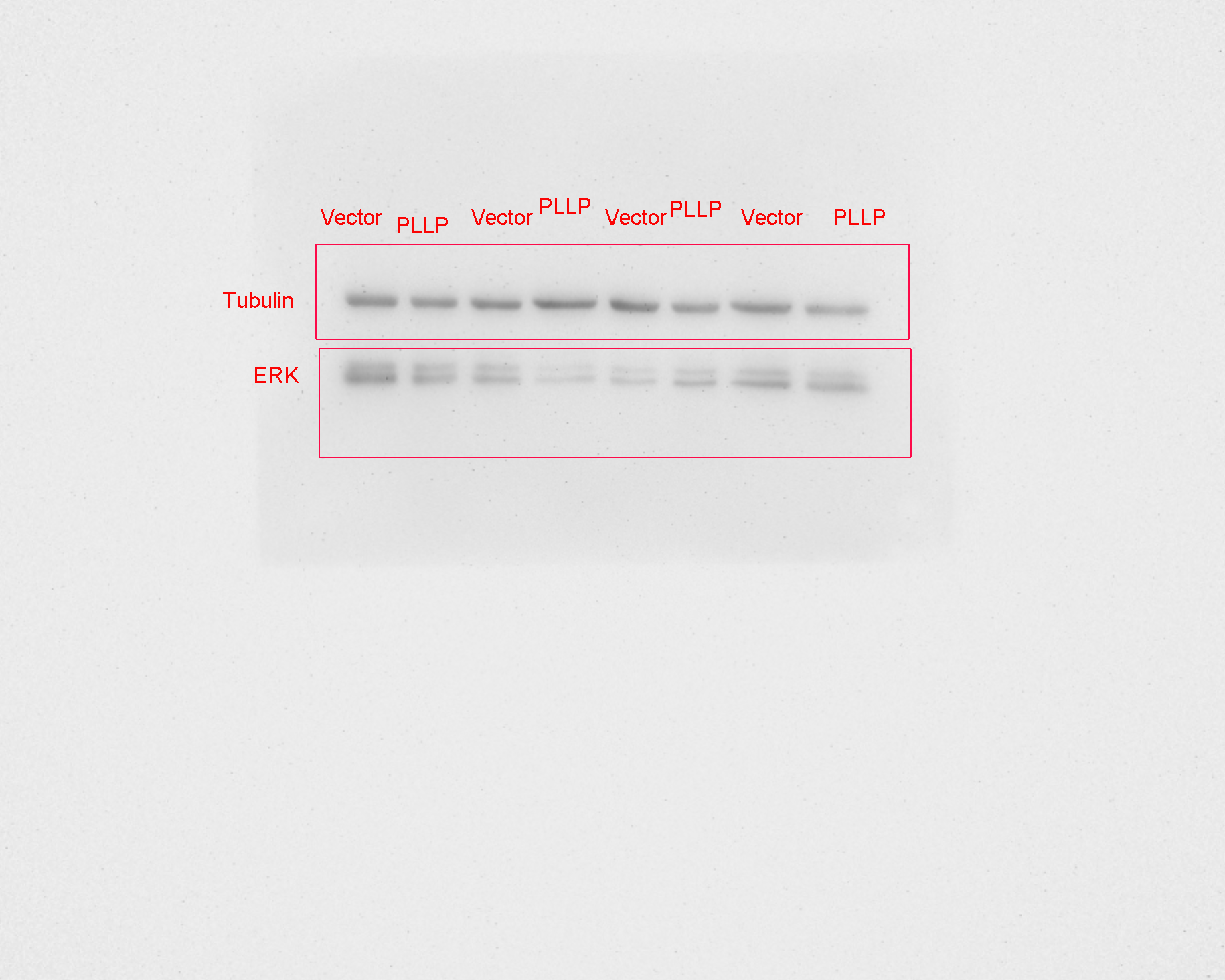

Supplement: Supplementary file 1 — Original Images-WB [file 41420_2025_2526_MOESM1_ESM.zip › Original Images-WB/FigS6C-ERK tubulin.tif]

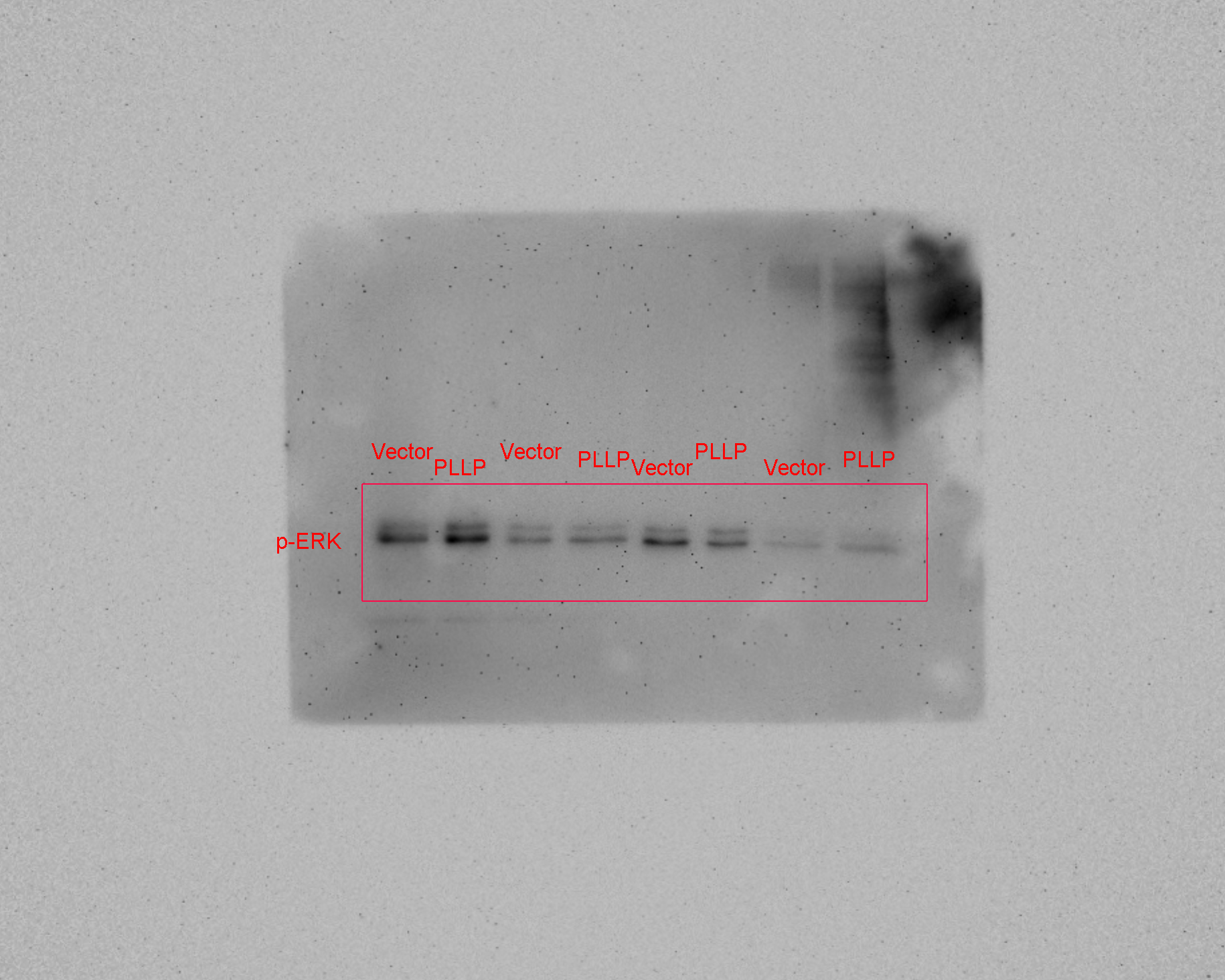

Supplement: Supplementary file 1 — Original Images-WB [file 41420_2025_2526_MOESM1_ESM.zip › Original Images-WB/FigS6C-pERK.tif]

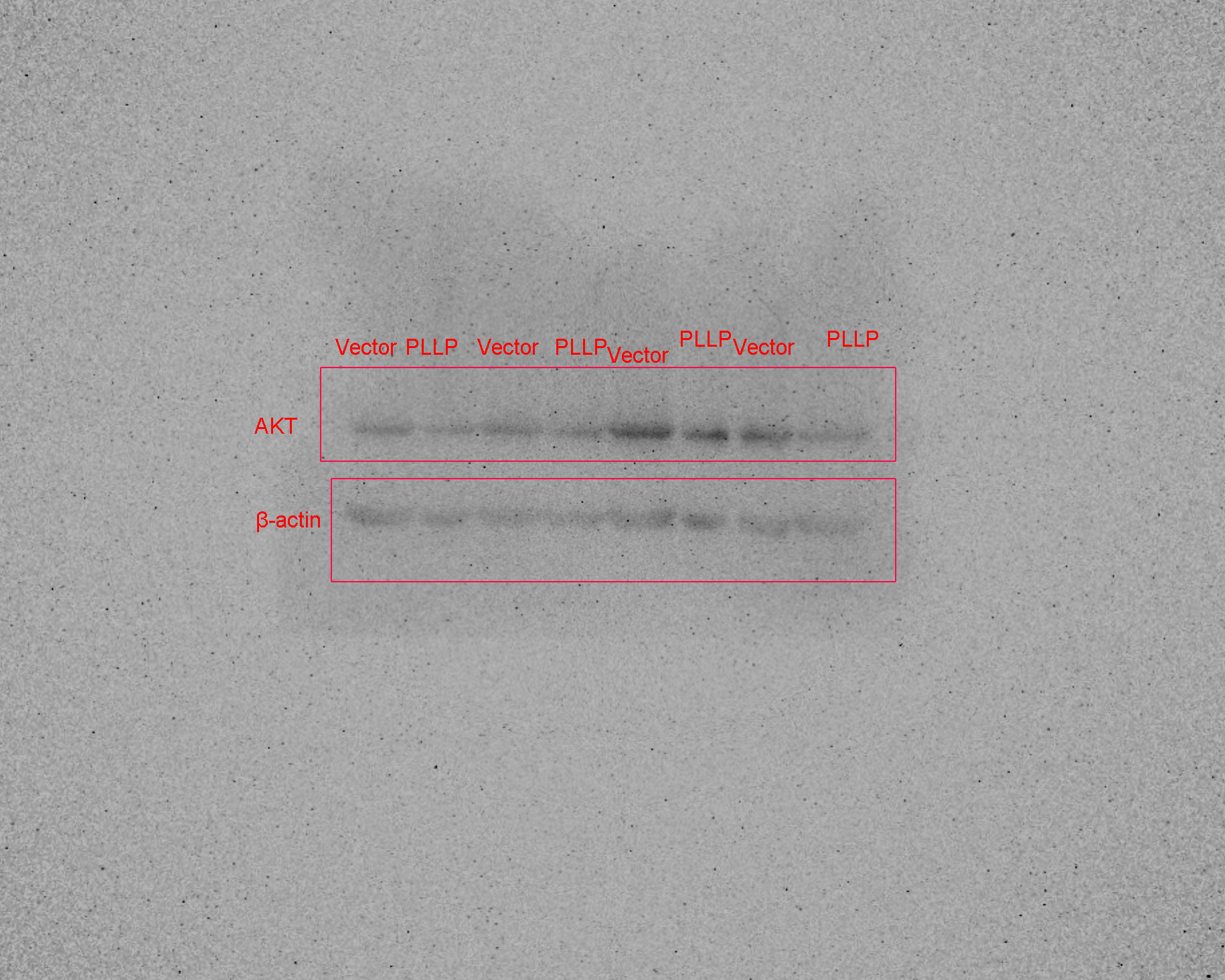

Supplement: Supplementary file 1 — Original Images-WB [file 41420_2025_2526_MOESM1_ESM.zip › Original Images-WB/FigS6D-AKT β-actin.tif]

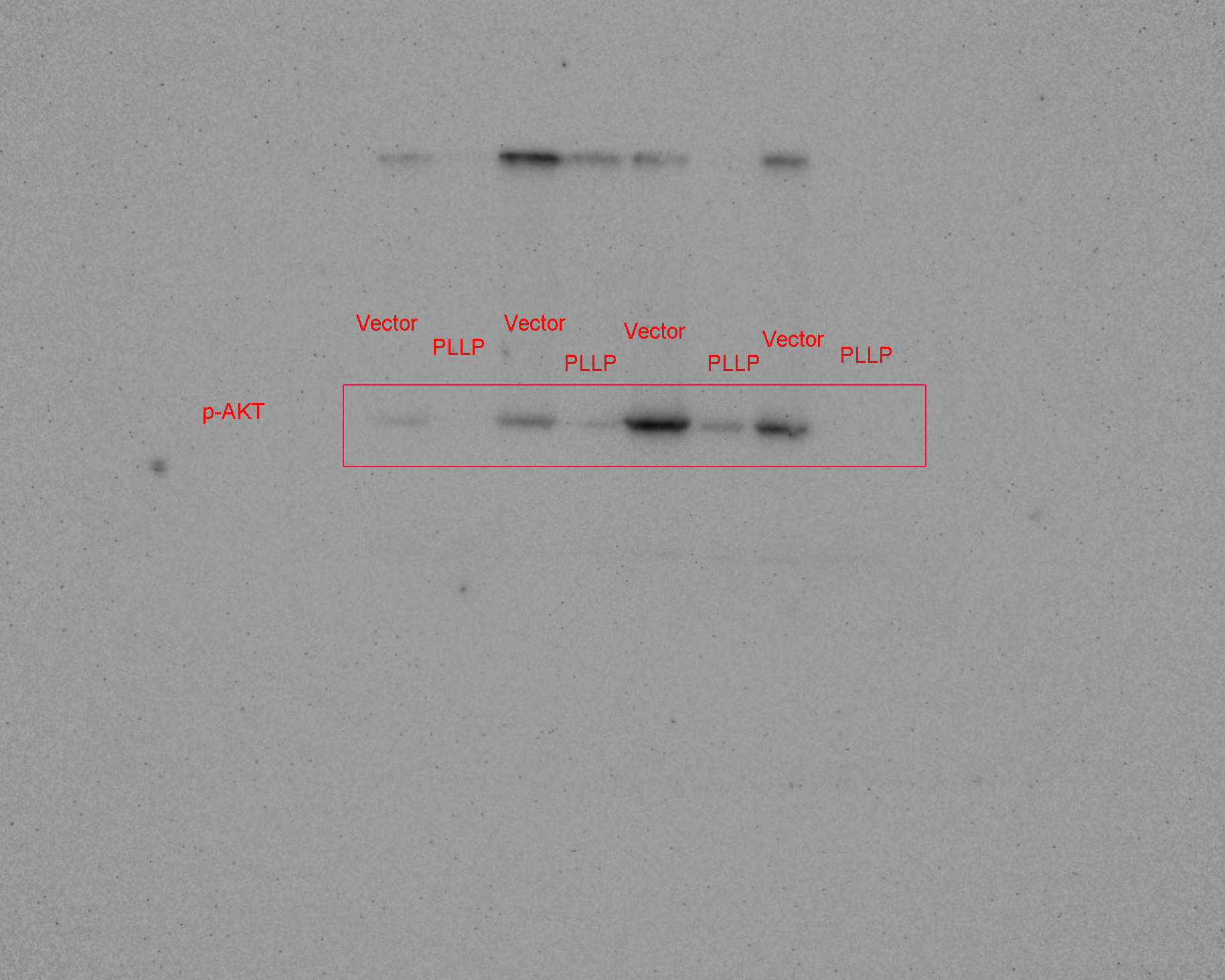

Supplement: Supplementary file 1 — Original Images-WB [file 41420_2025_2526_MOESM1_ESM.zip › Original Images-WB/FigS6D-pAKT.tif]
