# Supplementary material for: Plasmolipin deficiency is essential for HUVECs survival under hypoxic conditions
Source: Cell Death Discov. 2025 May 17;11:239. doi: 10.1038/s41420-025-02526-5 (PMC12084367; doi:10.1038/s41420-025-02526-5)
Supplement: Supplementary file 2 — Additional Information [file 41420_2025_2526_MOESM2_ESM.docx]

**ADDITIONAL INFORMATION**

**Supplementary information** Supplemental Figure Legend; Figure S1. Process of CRISPR/Cas9 screening for hypoxia related factors; Figure S2. Expression of PLLP protein in mice tissues evaluated by western blot; Figure S3. Knocking down of PLLP increased the cell proliferation of primary HUVECs in hypoxia; Figure S4. The invasion of HUVECs under hypoxic conditions after altering the expression of PLLP; Figure S5. Knocking out of PLLP increased the angiogenic ability of HUVECs in hypoxia; Figure S6. PLLP deficiency activated the AKT and ERK1/2 signaling pathways on HUVECs in hypoxia; Table S1. Primers for PCR detection.
