# Supplementary material for: Plasmolipin deficiency is essential for HUVECs survival under hypoxic conditions
Source: Cell Death Discov. 2025 May 17;11:239. doi: 10.1038/s41420-025-02526-5 (PMC12084367; doi:10.1038/s41420-025-02526-5)
Supplement: Supplementary file 3 — Supplement Table 1 Primers for PCR detection [file 41420_2025_2526_MOESM3_ESM.docx]

**Supplement Table 1** **Primers for PCR detection**

| Gene | Forward/Reverse | 5’ - 3’ |
| --- | --- | --- |
| Cas9 | Forward | TGGTGGAAGAGGATAAGAAGCA |
|  | Reverse | CAGGTCGTCGTCGTAGGTGTC |
| GAPDH | Forward | GAAGGTGAAGGTCGGAGTC |
|  | Reverse | GAAGATGGTGATGGGATTTC |
| sgRNA universal | Forward | AATGGACTATCATATGCTTACCGTAACTTGAAAGTATTTCG |
|  | Reverse | CTTTAGTTTGTATGTCTGTTGCTATTATGTCTACTATTCTTTCC |
| qPCR-PLLP | Forward | GTCTTCCTCTGGCTGGTGAC |
|  | Reverse | ACCAAACACGCAAAGAACGAG |
| qPCR- PL13 | Forward | TCAAAGCCTTCGCTAGTCTCC |
|  | Reverse | GGCTCTTTTTGCCCGTATGC |
| qPCR-VEGF | Forward | CCCACTGAGGAGTCCAACAT |
|  | Reverse | CGGCTTGTCACATTTTTCTTGT |
| qPCR-TGFB1 | Forward | GGAAATTGAGGGCTTTCGCC |
|  | Reverse | CCGGTAGTGAACCCGTTGAT |
| qPCR-ANGPT1 | Forward | GCTGAACGGTCACACAGAGA |
|  | Reverse | TTCCCCCTCAAAGAAAGCGT |
| qPCR-LAMA4 | Forward | GAGAAGGAAGCCAGGACAGC |
|  | Reverse | CTCTCCGTGTGCAGTATCCC |
| qPCR-SERPINE1 | Forward | AGAGCGCTGTCAAGAAGACC |
|  | Reverse | AGTTCTCAGAGGTGCCTTGC |
| qPCR-THBD | Forward | TGGGTAACATGCTTGGGGTC |
|  | Reverse | ACTGGCATTGAGGAAGGTCG |
| qPCR-ICAM1 | Forward | TCTTCCTCGGCCTTCCCATA |
|  | Reverse | AGGTACCATGGCCCCAAATG |
| qPCR-OCLN | Forward | CTCCCTGGCACCGTTGG |
|  | Reverse | GCCTGGATGACATGGCTGAT |
| qPCR-CDH5 | Forward | CCCACAGGCACGATCTGTT |
|  | Reverse | GTTGGGCAGGGTTAGCACC |
| qPCR-CLDN5 | Forward | GATTGAGAGGTCTGGGAAGCC |
|  | Reverse | ATCCCATGGCAAACAGAGAGG |
| qPCR-NOS3 | Forward | GTGTCCCTCGAACACGAGAC |
|  | Reverse | AGTGGGTCTGAGCAGGAGAT |
| qPCR-KDR | Forward | CGGTCAACAAAGTCGGGAGA |
|  | Reverse | CAGTGCACCACAAAGACACG |
| qPCR-ENG | Forward | CACTGCTGCACTCTGGTACA |
|  | Reverse | CCCTCAATCCCTCAGAGGCT |
| qPCR-VWF | Forward | GGCTTTATCTCCCCCAGCAG |
|  | Reverse | CCAAGGTCCCTGGGAAACTC |
| qPCR-Ms-AKT | Forward | GAGACCTTGGTGAGGAAGGAC |
|  | Reverse | TGGTGCACATTCATCCGTAAG |
| qPCR-Ms-MAPK | Forward | CTGCCTGGTGGACACTAACG |
|  | Reverse | TGTTGGCGGTACAGTCCTTAG |
