# Supplementary material for: Plasmolipin deficiency is essential for HUVECs survival under hypoxic conditions
Source: Cell Death Discov. 2025 May 17;11:239. doi: 10.1038/s41420-025-02526-5 (PMC12084367; doi:10.1038/s41420-025-02526-5)
Supplement: Supplementary file 4 — Supplemental Figure Legend [file 41420_2025_2526_MOESM4_ESM.docx]

**Fig. S1** **Process of CRISPR/Cas9 screening for hypoxia related factors.** **A** mRNA expression of endothelial-related genes in primary and immortalized HUVECs. **B** Expression of Cas9 in the genome after HUVEC transfection with lentiCas9. **C** Agarose gel electrophoresis assay to detect the mRNA expression of Cas9 after HUVEC transfection with lentiCas9. **D** Protein expression of CAS9 after HUVEC transfected lentiCas9. **E** The state of immortalized HUVECs and sgCtrl culture after 48 hours under normal and hypoxia conditions. NOR, cells cultured in normoxia. HY, cells cultured in hypoxia. sgCtrl, the negative control group of PLLP knockout cells. **F** Genomic sequencing alignment after transfection of PLLP-gRNA in HUVECs. **G** The sequencing results of the selected monoclonal cells transfecting to PLLP-gRNA show the changes in base. **H** EdU incorporation experiment to detect the cell proliferation ability of MOCK, sgPLLP and sgCtrl cells under 21% O_2_ and 6% O_2_ for 24 hours. MOCK, no transfected HUVECs. Blue represents DAPI, represents total cells, red represents EdU, represents proliferative cells. Scale bar, 25 μm. **I** mRNA expression of PLLP in HUVEC after transfected PLLP-gRNA. **J** Protein expression of PLLP after HUVEC transfected PLLP overexpressing plasmid. Vector, the negative control group of PLLP overexpressing cells. PLLP, the PLLP overexpressing cells. **K** mRNA expression of PLLP in HUVECs after 24 or 48h in hypoxia. The results are expressed as the mean ± standard deviation and analyzed using a two-tailed Student's t-test (n=3, *p<0.05, **p<0.01, ***p<0.001, ns=not significant.).

**Fig. S2** **Expression of PLLP protein in mice tissues evaluated by western blot.** **A** **and** **B** Protein expression of PLLP in kidney, stomach, lung, intestines, heat, brain, spleen, testis or ovary of male or female mice.

**Fig. S3** **Knocking down of PLLP increased the cell proliferation of primary HUVECs in hypoxia.** **A** mRNA expression of PLLP in primary HUVECs after transfected siRNA. Scramble, the negative control group of PLLP knockdown cells. siPLLP, the PLLP knockdown cells. **B** EdU incorporation experiment to detect the cell proliferation ability of MOCK, siPLLP and Scramble primary HUVECs under normoxia and hypoxia for 24 hours. Blue represents DAPI, represents total cells, green represents EdU, represents proliferative cells. Scale bar, 25 μm. **C** Quantify the basal respiration, maximal respiration and non-mitochondrial oxygen consumption in Fig.2G. **D** Quantify the basal respiration, maximal respiration and non-mitochondrial oxygen consumption in Fig.2H. The results are expressed as the mean ± standard deviation. **A**, **C**, **D** analyzed using a two-tailed Student's t-test and **B** analyzed using Two-way ANOVA (n=3, *p<0.05, **p<0.01, ***p<0.001, ns=not significant.).

**Fig. S4** **The invasion of HUVECs under hypoxic conditions after altering the expression of PLLP.** **A** Transwell invasion assays to detect the invasion ability of HUVECs after knocking out PLLP under normal and hypoxia conditions. **B** Transwell invasion assays to detect the invasion ability of HUVECs after overexpressing PLLP under normal and hypoxia conditions. The results are expressed as the mean ± standard deviation and analyzed using Two-way ANOVA (n=3, *p<0.05, **p<0.01, ***p<0.001, ns=not significant.).

**Fig. S5** **Knocking out of PLLP increased the angiogenic ability of HUVECs in hypoxia. A-C** Quantify the number branches, total segments lenght and total meshes area displayed in Fig.4A using Image J. **D-F** Quantify the number branches, total segments lenght and total meshes area displayed in Fig.4D using Image J. The results are expressed as the mean ± standard deviation and analyzed using Two-way ANOVA (n=3, *p<0.05, **p<0.01, ***p<0.001, ns=not significant.).

**Fig. S6 PLLP deficiency activated the AKT and ERK1/2 signaling pathways on HUVECs in hypoxia A** mRNA expression of Akt in cerebral ischemia models (n=6). **B** mRNA expression of MAPK(Erk) in cerebral ischemia models (n=6). **C** Expression of ERK1/2 and phospho-ERK1/2 proteins evaluated by western blot in HUVECs overexpressing PLLP that treating with U0126 or not and cultivated for 24 hours in normoxia or hypoxia. Quantification of western blot with Image J. **D** Expression of AKT and phospho-AKT proteins evaluated by western blot in HUVECs overexpressing PLLP that treating with LY294002 or not and cultivated for 24 hours in normoxia or hypoxia. Quantification of western blot with Image J. The results are expressed as the mean ± standard deviation. **A**, **B** analyzed using a two-tailed Student's t-test and **C**, **D** analyzed using Two-way ANOVA (n=3, *p<0.05, **p<0.01, ***p<0.001, ns=not significant.).
